# Azobenzenesulfonamide Carbonic Anhydrase Inhibitors as New Weapons to Fight *Helicobacter pylori*: Synthesis, Bioactivity Evaluation, In Vivo Toxicity, and Computational Studies

**DOI:** 10.3390/ph17081027

**Published:** 2024-08-05

**Authors:** Letizia Giampietro, Beatrice Marinacci, Alice Della Valle, Ilaria D’Agostino, Aldo Lauro, Mattia Mori, Simone Carradori, Alessandra Ammazzalorso, Barbara De Filippis, Cristina Maccallini, Andrea Angeli, Clemente Capasso, Santolo Francati, Adriano Mollica, Rossella Grande, Claudiu T. Supuran

**Affiliations:** 1 Department of Pharmacy, “G. d’Annunzio” University of Chieti-Pescara, 66100 Chieti, Italy; beatrice.marinacci@unich.it (B.M.); alice.dellavalle@unich.it (A.D.V.); simone.carradori@unich.it (S.C.); alessandra.ammazzalorso@unich.it (A.A.); barbara.defilippis@unich.it (B.D.F.); cristina.maccallini@unich.it (C.M.); adriano.mollica@unich.it (A.M.); rossella.grande@unich.it (R.G.); 2 Department of Innovative Technologies in Medicine & Dentistry, “G. d’Annunzio” University of Chieti-Pescara, 66100 Chieti, Italy; 3 Department of Pharmacy, University of Pisa, Via Bonanno 6, 56126 Pisa, Italy; ilaria.dagostino@unipi.it; 4 Department of Biotechnology, Chemistry and Pharmacy, University of Siena, Via Aldo Moro 2, 53100 Siena, Italy; aldo.lauro@outlook.it (A.L.); mattia.mori@unisi.it (M.M.); 5 Neurofarba Department, University of Florence, Sesto Fiorentino, 50019 Florence, Italy; andrea.angeli@unifi.it (A.A.); claudiu.supuran@unifi.it (C.T.S.); 6 Department of Biology, Agriculture and Food Sciences, National Research Council (CNR), Institute of Biosciences and Bioresources, 80131 Naples, Italy; clemente.capasso@ibbr.cnr.it; 7 Department of Agricultural and Food Sciences (DISTAL), University of Bologna, 40126 Bologna, Italy; santolo.francati2@unibo.it; 8 Center for Advanced Studies and Technology, “G. d’Annunzio” University of Chieti-Pescara, 66100 Chieti, Italy

**Keywords:** azoderivatives, carbonic anhydrase, *Helicobacter pylori*, *Galleria mellonella*, phenyldiazenyl, sulfonamide

## Abstract

Research into novel anti-*Helicobacter pylori* agents represents an important approach for the identification of new treatments for chronic gastritis and peptic ulcers, which are associated with a high risk of developing gastric carcinoma. In this respect, two series of azobenzenesulfonamides were designed, synthesized, and tested against a large panel of human and bacterial CAs to evaluate their inhibitory activity. In addition, computational studies of the novel primary benzenesulfonamides (**4a**–**j**) were performed to predict the putative binding mode to both HpCAs. Then, the antimicrobial activity versus *H. pylori* of the two series was also studied. The best-in-class compounds were found to be **4c** and **4e** among the primary azobenzenesulfonamides and **5c** and **5f** belonging to the secondary azobenzenesulfonamides series, showing themselves to exert a promising anti-*H. pylori* activity, with MIC values of 4–8 μg/mL and MBCs between 4 and 16 μg/mL. Moreover, the evaluation of their toxicity on a *G. mellonella* larva in vivo model indicated a safe profile for **4c**,**e** and **5c**,**f**. The collected results warrant considering these azobenzenesulfonamides as an interesting starting point for the development of a new class of anti-*H. pylori* agents.

## 1. Introduction

Counteracting *Helicobacter pylori* infection is crucial for Public Health worldwide [1,2]. This gastroduodenal pathogen is associated with chronic gastritis and peptic ulcers, as well as a higher risk for people affected with developing gastric carcinoma [3,4,5]. However, the human stomach is not the only *H. pylori* ecological niche: pieces of evidence show that this bacterium can be detected in both human saliva and the esophagus, suggesting different potential means of transmission [6,7]. Also, the increasing resistance and tolerance of the bacterium to the most commonly used antimicrobial are of particular concern [8,9]. The former is associated with genetic variability, whereas the latter is supported by two main phenotypic modifications which are the switch from spiral to coccoid morphology, extracellular vesicles secretion, and biofilm formation [10,11,12]. In 2017, the World Health Organization included *H. pylori* in the high-priority category of drug-resistant bacteria, encouraging research in this field and the development of new antimicrobials. Considering this critical issue, the identification of novel targets among bacterial proteins or enzymes is highly recommended, representing a promising strategy [13,14]. Previously, we demonstrated that targeting *H. pylori* Carbonic Anhydrases (HpCAs) may represent a good option for the treatment of infections sustained by the pathogen’s drug-resistant strains. These enzymes are involved in the adaptation of the microorganism to the acidic environment of the stomach, affecting its ability to survive and colonize tissues [15]. Further studies also highlighted the ability of CA inhibitors to prevent *H. pylori* biofilm formation from interfering with the release and extracellular (e)DNA content of outer membrane vesicles (OMVs) [16,17].

**Figure 1:**
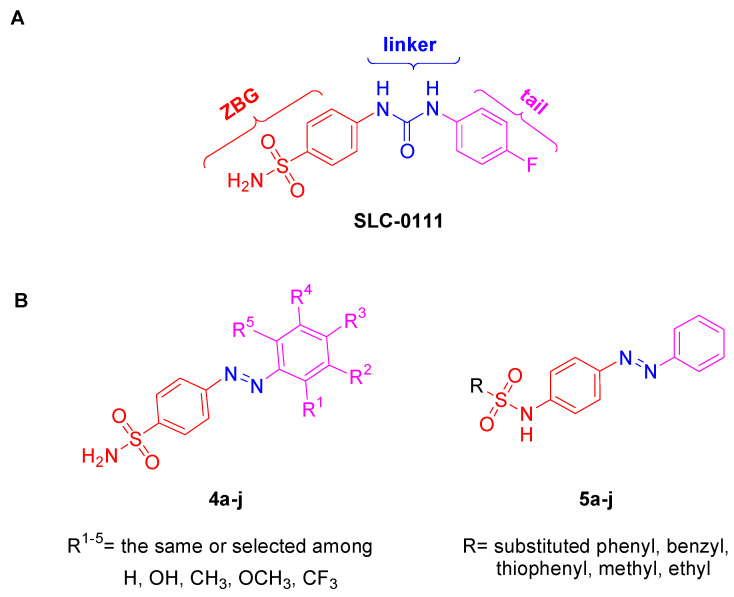
(**A**) Molecular structure of SLC-0111; (**B**) general structures of compounds belonging to primary azobenzenesulfonamide (**4a**–**j**) and reversed secondary azobenzenesulfonamide (**5a**–**j**) series. Specific substitution patterns are reported in Scheme 1, Table 1 and Table 2.

Sulfonamide-based compounds have shown different biological activities such as aromatase inhibition [18] and anticonvulsant, anticancer, antibacterial [19], antiviral, antimalarial, antifungal, and antidepressant effects [20,21]. In particular, aromatic primary sulfonamide derivatives are known to exert strong CA inhibitory effects, especially because the sulfonamide moiety acts as a zinc-binding group (ZBG) and the aromatic group promotes the interactions with residues close to the active site of the different CA isoforms [22,23]. In the last years, CA inhibitor (CAI) research has referred to the discovery of isoform-selective sulfonamide inhibitors using ring and tail approaches. In the first method, the ring connected to the ZBG is modulated, changing its chemical nature, whereas the tail approach consists of constructing a specific moiety on the ZBG ring in order to allow specific interactions with the amino acids far away from the active site, providing for isoform selectivity. An example is given by SLC-0111 (Figure 1A), a selective human (*h*) CA IX/XII inhibitor that is in clinical trials for the treatment of metastatic solid tumors or as an adjuvant in pancreatic ductal carcinoma.

Based on the above-mentioned findings and keeping in mind the importance of this enzyme in biomedical applications [24], the goal of this work was the synthesis of new CAIs, with the aim of understanding how the chemical modification of the structure of classical CAIs could influence the selectivity and the anti-*H. pylori* effect of these compounds. In particular, we synthesized new azobenzenesulfonamides by using at first the tail approach, obtaining a series of compounds replacing the ureidic group between the ZBG and the tail, with an azo linker often used in medicinal chemistry as bioisosteres of the double carbon bond of the stilbene moiety [25,26]. In these compounds, the tail was systematically changed, introducing substituents with different electronic effects and steric hindrance, in different positions of the aromatic ring (**4a**–**j**, Figure 1B). In order to better comprehend the role of the *para*-diazenylphenyl tail, we also took into consideration a previously synthesized series of secondary reversed azobenzenesulfonamides (**5a**–**j**, Figure 1B), in which the sulfonamide moiety was functionalized with different aromatic, heteroaromatic, or alkyl chains [18].

In the present study, the target molecules were evaluated against a panel of human and bacterial CAs to evaluate their inhibitory activity and isoform/family selectivity, using acetazolamide (**AAZ**) (Table 1), a well-known CA inhibitor, as a reference drug. Moreover, computational studies of the new primary azobenzenesulfonamides (**4a**–**j**) were performed to predict the putative binding mode on both HpCAs. Finally, the antimicrobial activity versus *H. pylori* of the two series and the in vivo toxicity versus *Galleria mellonella* of the most promising compounds were also assessed.

## 2. Results and Discussion

### 2.1. Chemistry

**Scheme 1:**
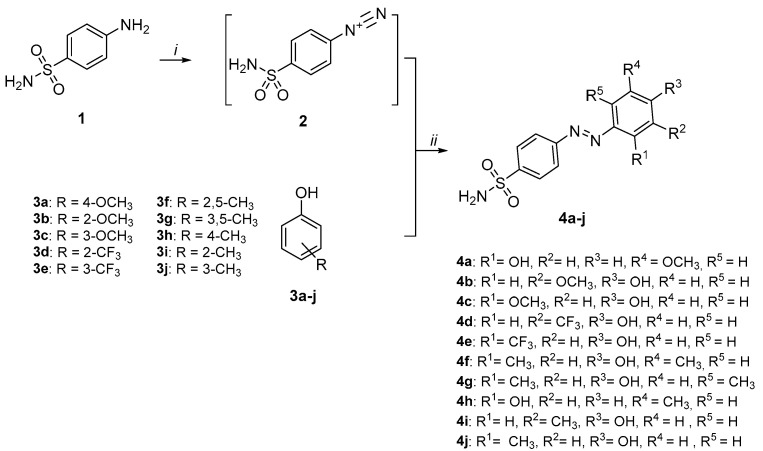
Synthesis of primary azobenzenesulfonamides **4a**–**j**. Reagents and conditions: (i) NaNO_2_, 6N HCl, 0–5 °C, 1 h; (ii) 4N NaOH, 0–5 °C 4 h.

The synthetic strategy for the synthesis of the primary azobenzenesulfonamides is reported in Scheme 1. 4-Aminobenzenesulfonamide **1** was converted into diazonium salt **2** through sodium nitrite and hydrochloric acid at 0–5 °C and then, the suitable phenol **3a**–**j** was added in a basic environment to give azoderivatives **4a**–**j** (Scheme 1) [27].

To better understand the role of the *para*-diazenylphenyl tail in the in vitro inhibitory assays, we also considered a previously synthesized series of secondary reversed azobenzenesulfonamides (**5a**–**j**, Table 2) functionalized at the *para* position with the same chemical fragments [18].

### 2.2. Inhibitory Activity towards a Panel of Carbonic Anhydrases and Structure–Activity Relationships

Compounds **4a**–**j** and **5a**–**j** were tested on a large panel of human and bacterial isoenzymes belonging to different CA families, evaluating their inhibitory activity through the CO_2_ hydration activity assay by means of the stopped-flow technique, and the inhibitory constants (*K*_I_s) are reported in Table 1 and Table 2, respectively. In particular, we tested the hypoxic tumor-associated *h*CAs IX and XII, and bacterial isoforms involved in several infectious diseases, such as those caused by *H. pylori* (HpαCA and HpβCA), *Porphyromonas gingivalis* (PgiβCA and PgiγCA), *Escherichia coli* (EcoβCA), *Pseudomonas aeruginosa* (PsCA3, belonging to the β-class), *Mammaliicoccus* (*Staphylococcus*) *sciuri* (MscCA, belonging to the β-class), and *Streptococcus mutans* (SmuCA, belonging to the β-class). The inhibition of human physiologically relevant isoforms I and II was also investigated in order to assess the compounds’ selectivity. Our main aim was to rationalize the structural requirements of sulfonamide-containing compounds useful for targeting HpCAs, in order to gain a larger knowledge for further optimization studies focusing on isoform selectivity. In fact, although the tested human and bacterial isoforms present a low degree of sequence similarity, they catalyze the same reaction and contain the catalytically active zinc ion in the active site. Thus, a comparison of the data obtained from different members of this large superfamily has a high relevance in the field. By observing Table 1 and Table 2, interesting structure–activity relationship (SAR) considerations can be inferred.

Collectively, all the primary sulfonamides **4a**–**j** were found to inhibit human CAs in the nanomolar range, while a more selective inhibitory trend could be noticed when observing data on bacterial isoenzymes (Table 1). As regards the inhibitory activity towards human CAs, among the hydroxyanisoles **4a**–**c**, the presence of a hydroxy (-OH) function in the *ortho* position (**4a**) seems to address the selectivity towards *h*CA XII over the other human isoforms, with a *K*_I_ value of 9.5 nM, while when it is in the *para* position (**4b** and **4c**) a more potent inhibition of *h*CA II, with *K*_I_ values of 3.3 and 5.4 nM, respectively, is highlighted. The presence and the position of a trifluoromethyl substitution on the phenol ring significantly affect the selectivity, with **4d** showing a preference for *h*CA II and **4e** for *h*CAs I and II, inhibiting these isoenzymes at low nanomolar concentrations (*K*_I_s = 4.8 and 5.4–4.2 nM, respectively). The same trend is observed for dimethyl phenols **4f** and **4g**. In these cases, they showed a preference for *h*CAs I and II, with *K*_I_ values of 5.7 (*h*CA I) and 1.9 nM (*h*CA II) for **4f** and *K*_I_s equal to 4.9 and 3.7 nM on *h*CAs I and II, respectively, for **4g**. The cresols **4h**–**j** display a different inhibitory profile. In fact, while **4h** and **4i** show a notable potency in inhibiting *h*CA XII (*K*_I_s = 9.6 and 7.5 nM, respectively), even if in a non-selective manner, compound **4j** is less potent against this isoform, exerting a medium nanomolar inhibition (*K*_I_s = 84.1 nM). Conversely, **4h**, **4i**, and **4j** show a good inhibition of *h*CA II, with *K*_I_ values of 6.6, 3.4, and 4.7 nM, respectively. The formers, compounds **4i** and **4j,** show a notable inhibition also against *h*CA I, with *K*_I_ values of 4.4 and 5.3 nM, respectively. By analyzing these data, it is possible to assume that, for derivatives **4a**–**c**, the position of the substituent notably influences the selectivity, whereas this behavior is not maintained in compounds **4a**–**j**, where a good inhibition of both *h*CAs I and II was noticed, except for compounds **4d**, **4h,** and **4i**. The trifluoromethyl substitution in the *ortho* position on the phenol ring (**4d**) provided a higher selectivity for *h*CA II, whereas the methyl substitution in *para* (**4h**) and *ortho* (**4i**) on the phenol ring also corresponded to a good inhibition against *h*CA XII.

The other half part of Table 1 reports *K*_I_ values on bacterial CAs that belong to different classes (α, β, or γ). All the compounds turned out to be more potent against the two CAs expressed by *H. pylori* over the other bacterial isoenzymes, thereby showing an isoform preference or selectivity.

The data on *P. gingivalis* CAs (PgiβCA and PgiγCA) show a high nanomolar/low micromolar inhibition for primary sulfonamides **4a**–**j** (Table 1). In particular, analyzing enzymatic assay results on the β isoform, in the hydroxyanisoles **4a**–**c** series, the presence of two (**4f**,**g**) or one (**4h**–**j**) methyl substituents weakens the inhibitory potency, reporting *K*_I_ values in the range of 3.6–5.7 µM. A different trend is recorded on the γ-CA: the methoxy group in *ortho* to the phenolic function (**4b**) maintains the inhibitory activity in the high nanomolar range, while the presence of the trifluoromethyl substitutent in the same position in **4d** causes a remarkable inhibition of the enzyme, with a low nanomolar *K*_I_ (=94.7 nM) which is 3.5-fold lower than that reported for the isomer **4e**; no interesting activity profile emerges for the mono- or di-methylphenol derivatives **4f**–**j**, all showing high nanomolar *K*_I_s, with **4i** reaching a low micromolar activity (*K*_I_ = 6.3 µM).

As anticipated, the inhibitory activities on HpCAs are very interesting and put the spotlight on this bacterium for further investigation (Table 1). In fact, observing the *K*_I_s values for HpαCA, we can notice a similar activity for **4a** and **4c** (*K*_I_s = 65.8 and 70.9 nM, respectively) and a slightly worsened one for **4b** (*K*_I_ = 120 nM). Also, the trifluoromethyl group decreases the *K*_I_ to 39.0 nM when the substituent is in *ortho* with respect to the phenolic function in **4d**; in contrast, the methyl group is better tolerated in the substitution pattern of **4f** and **4j** (*K*_I_s = 69.4 and 72.2 nM, respectively) compared to the others (**4g**–**i**, *K*_I_s = 130–142 nM). It is possible to notice that the substitution with trifluoromethyl in the *ortho* position to the OH group gives the best *K*_I_ value for HpαCA. However, the SARs are quite different for HpβCA, with **4c** and **4d** reaching the very low *K*_I_ values of 17.1 and 19.4 nM, respectively, also lower than the reference compound **AAZ** (*K*_I_ = 40 nM). In these compounds, the introduction of a methoxy (**4c**) and a trifluoromethyl (**4d**) group in the *meta* and *ortho* positions leads to the best activity. Conversely, the other patterns of methoxy (**4a**,**b**), trifluoromethyl (**4e**), and methyl (**4f**–**j**) substitutions can still record interesting inhibitory profiles, even if less potent in the case of methylphenols **4h**–**j** (*K*_I_s = 119–138 nM).

*K*_I_s on *E. coli* CA from the β-class range from low nanomolar to low micromolar concentrations, with the lowest values shown by **4b**, **4c**, and **4e** (*K_I_* = 95.3–96.3 nM), sharing the common feature of bearing the phenolic group in *para* to the azo function (Table 1). Otherwise, the presence of a methyl substituent in *ortho* or *para* to the phenolic function does not seem to be tolerated, conferring to compounds **4i**,**j** the lowest potency at a micromolar level (*K*_I_s = 2.1 and 3.0 µM, respectively).

Moreover, these primary sulfonamides are able to inhibit PsCA3 from the β-class at low–high nanomolar concentrations (Table 1). Analyzing data for each group of isomers, there are differences between *K*_I_ values of about 3-fold for **4a**–**c**, 2-fold for compounds **4d**,**e**, and about 5-fold for **4f**,**g**. Two compounds emerge for their *K*_I_ values: the methylphenols **4i** and **4j**, being two isomers with more than a 10-fold difference in potency (*K*_I_s = 114 and 1845 nM, respectively). In particular, the presence of the methyl function in *meta* to the phenol, peculiar to **4j**, seems to be better tolerated if the substitution pattern also involves a second methylation in the *meta* (**4g**, *K*_I_ = 897 nM) or in the *ortho* (**4f**, *K*_I_ = 191 nM) position.

The inhibition of another β-CA, MsCA, occurs at micromolar concentrations and no relevant SARs can be performed since the range is very narrow for all the compounds (*K*_I_s = 5.3–7.7 µM), with the exception for lower values recorded by hydroxyanisoles **4a** and **4b** (*K*_I_s = 2.9 and 2.8 µM, respectively).

In the end, β-CA from *S. mutans* is assumed to be highly responsive to the small chemical modifications of the compounds, being inhibited with a *K*_I_ value of 82.1 nM by methylphenol **4h**, which increases 2.5/6-fold in the case of isomerization (**4i**,**j**) or the addition of another methyl substituent on the phenol ring (**4f**,**g**).

The second set of derivatives (secondary benzenesulfonamides **5a**–**j**), previously published for other purposes, were added only to extrapolate more robust SARs in the context of sulfonamide-containing CA inhibitors and to explore a possible repositioning as antibacterial agents. Indeed, sulfonamides are usually potent CA inhibitors but endowed with quite low selectivity, even if the tail approach seems to be effective in modulating the compound’s preference for a specific isoform or class of isoenzymes. The secondary azobenzenesulfonamides **5a**–**j**, as expected, show a lower inhibitory potency compared to the primary ones **4a**–**j**, reporting *K*_I_s ranging from medium-high nanomolar to high micromolar values (Table 2).

Observing data on human CAs, we can notice that only two compounds, **5c** and **5i**, are active on *h*CA II, even if at high concentrations (*K*_I_s = 24.7 and 97.0 µM, respectively), while different trends of inhibition can be found on *h*CAs I, IX, and XII. In particular, focusing the attention on *h*CA I, the unsubstituted phenyl ring of **5a** causes the best activity in the series (*K*_I_ = 209 nM), while the introduction of cyano and nitro substituents dramatically worsen the inhibitory data (*K*_I_s = 359 and 6749 nM for **5b** and **5d**, respectively). Double substitution patterns on the phenyl ring (**5c**,**e**,**f**) are also detrimental and increase the *K*_I_ values to the high nanomolar range (*K*_I_s = 853–908 nM). Thienyl **5g** and benzyl **5h** do not improve the inhibitory data, while switching from the methyl substituent of **5i** to the ethyl one of **5j,** a great increase in potency (6.4-fold) can be observed. Surprisingly, cyano and nitro substitutions in **5b** and **5d**, respectively, improve the activity towards *h*CAs IX and XII. However, the introduction of a chlorine atom on the cyanophenyl ring in **5c** leads to an increase in *K*_I_ values for both the isoenzymes, while the methyl substitution on the nitrophenyl ring in compound **5e** worsens the inhibitory activity compared to nitrophenyl derivative **5d** but still maintains a higher potency compared to the unsubstituted phenyl **5a**. Instead, micromolar inhibition of *h*CAs IX and XII is recorded for thienyl **5g**, benzyl **5h**, and ethyl **5j**, while different results are reported for methyl compound **5i**, which represents the best in class of the series against *h*CA IX (*K*_I_ = 599 nM) and no worthy results of interest against *h*CA XII in terms of potency (*K*_I_ = 2.2 µM).

Also for this series, the second half of Table 2 reports *K*_I_s on bacterial CAs. Compounds **5a**–**j** prove to be inactive in inhibiting both the isoenzymes of *P. gingivalis* (PgCAs), with the exception of just one compound, the cyanophenyl **5b**, which shows a high micromolar inhibition of PgiβCA (*K*_I_ = 71.4 µM). Interesting activities on HpαCA emerge by observing the data in Table 2. In particular, the substitution of the phenyl ring always improves the inhibitory activity; in these cases, the *K*_I_ value passes from 460 nM for **5a** to 70–77 nM for **5b**–**d**, with the exception of compound **5e** (*K*_I_ = 604 nM). However, the *K*_I_ values are very close to each other and do not allow the performance of robust SARs. In this case, thienyl **5g** is the most potent compound of the series (*K*_I_ = 55.8 nM), although the other compounds are low nanomolar inhibitors of HpαCA. This good efficiency, especially of compounds **5a**–**d** and **5f**–**j**, is an interesting result considering their selectivity against the human CAs. Obtaining CAIs with selectivity against bacterial over human enzymes could be fundamental to avoid side effects.

Otherwise, micromolar activities are detected on EcoβCA (Table 2), and any kind of modification with respect to the phenyl ring of **5a** causes a dramatic decrease in the inhibitory potency. PsCA3 proves to be slightly inhibited by secondary azobenzenesulfonamides **5a**–**j,** and the *K*_I_ value of **5a** (*K*_I_ = 9.2 µM) is nearly decreased 2-fold by introducing the cyano substituent (**5b**, *K*_I_ = 5.7 µM) and increased 10-fold by the nitro function (**5d**, *K*_I_ = 90 µM). The disubstitution patterns of **5c**,**e**,**f** are not tolerated, as for **5g** and **5h**. Among the compounds bearing alkyl substituents, methyl **5i** shows a comparable activity to **5a**, whereas ethyl **5j** significantly lowers the potency (*K*_I_ = 69 µM). The relevant results are reported for MscCA: compounds **5a**–**j** exert a micromolar inhibition of such isoenzymes, with only the cyano derivative **5b** and the nitrotolyl compound **5e** reporting *K*_I_s in the low micromolar range (*K*_I_ = 6.9 and 8.5 µM, respectively), being the only derivatives worthy of note.

### 2.3. Computational Studies of Primary Azobenzenesulfonamides **4a**–**j** on Both HpCAs

**Figure 2:**
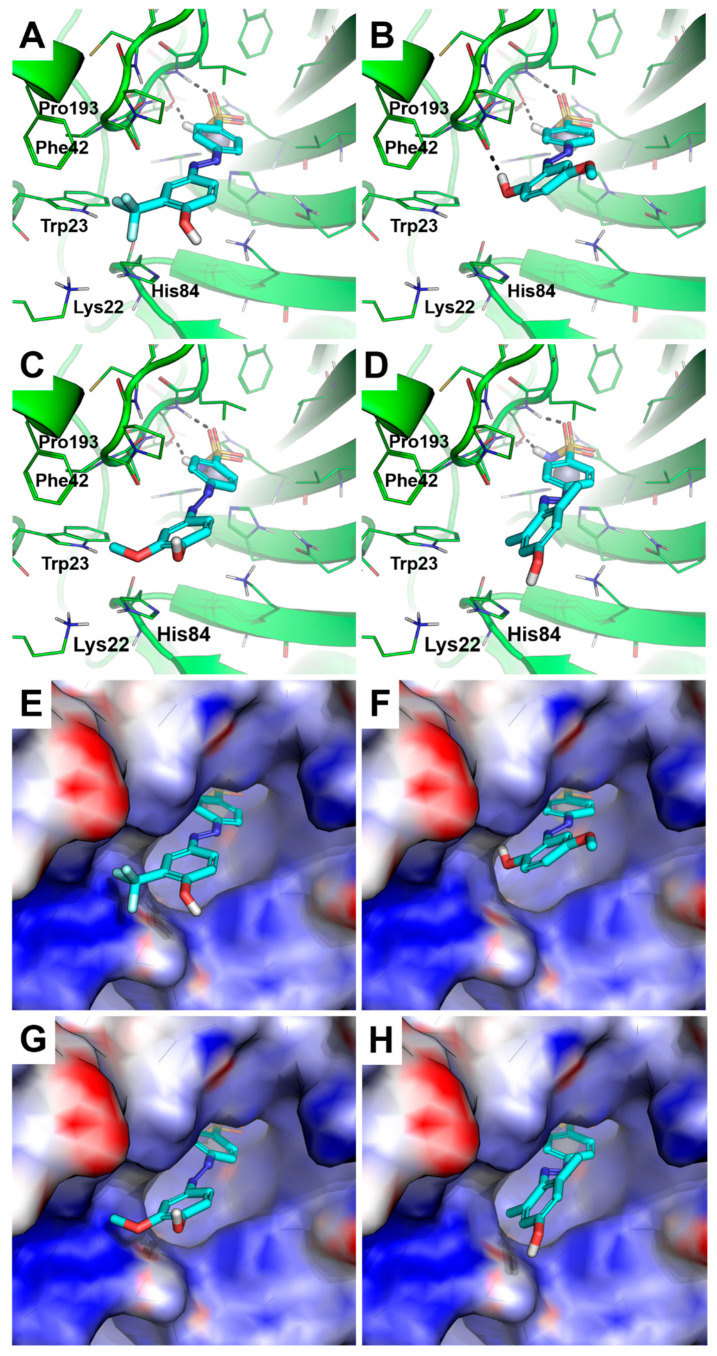
Predicted binding mode of compounds **4d** (**A**), **4a** (**B**), **4b** (**C**), and **4g** (**D**). The crystallographic structure of HpαCA, coded by PDB ID 4XFW, is shown as the green cartoon and lines in panels (**A**–**D**), while the protein surface is colored according to the electrostatic surface potential in panels (**E**–**H**) (blue = positive charge, red = negative charge, white = uncharged lipophilic regions). Residues within 4 Å from the ligands are shown as green lines. Those of **4d**,**a**,**b**,**g** are shown as cyan sticks; non-polar hydrogen atoms are omitted. The catalytic Zn(II) ion is shown as a gray sphere. Polar interactions between the inhibitors and HpαCA are highlighted by black dashed lines.

In order to study the possible binding mode of the ten new inhibitors **4a**–**j** against the two isoforms HpαCA and HpβCA and to provide structural support to a SAR analysis, molecular modeling studies were carried out. These two isoenzymes were the most inhibited among the several isoforms tested in this work (Table 1). While the crystallographic structure of HpαCA is available in the Protein Data Bank (PDB ID: 4XFW) [28], HpβCA’s structure has not been solved yet, and the generation of a homology model was required for the structure-based study described herein. Given the absence of structural details on the binding mode of **4a**–**j** (or close analogues of them) within the two HpCA isoforms, **4a**–**j** were docked by a non-covalent approach, using the deprotonated form of the sulfonamide zinc-binding group, with the aim to avoid, the imposition of conformational restraints and to mimic the well-known binding mode of sulphonamides to the catalytic Zn(II) ion of CAs. An analysis of the docking results on both HpCA isoforms gave insights into the SAR, and it was conducted by considering the molecules as composed of two portions: (i) a sulfonamide head and (ii) an aromatic tail. Docking against the HpαCA showed the head portion of **4a**–**j** binding to the catalytic Zn(II) ion. Besides Zn(II) coordination, the sulfonamide moiety establishes the canonical H-bond interactions with Thr191 (the residue numbering is in agreement with the crystallographic structure). The phenyl ring linked to the sulfonamide moiety occupies a position that is highly comparable with the available structural data (i.e., the crystallographic structure of HpαCA in complex with **AAZ**) [28]. The tail portion interacts in the variable region of the HpαCA within a cleft composed of Lys22, Trp23, Phe42, His84, Ala192, Pro193, and Pro194. Notably, the binding mode and orientation of the tail portion provide useful hints for a SAR analysis. Indeed, **4d** is one of the most potent inhibitors of both HpCA isoforms (*K*_I_ values = 39.0 nM and 19.4 nM against HpαCA and HpβCA, respectively). In HpαCA, the phenol tail is oriented towards the entrance of the catalytic site, and the trifluoromethyl group is docked in close proximity to the Lys22, His84, and Trp23 side chains (Figure 2A), which form a surface region endowed with a high density of positive charge (Figure 2E). The -OH group is exposed to the solvent.

Based on the docking simulations, the interaction between a polar moiety of the inhibitor and the positively charged cleft is conceived as an important feature for the strong inhibition of HpαCA. In fact, **4a** is a potent HpαCA inhibitor (*K*_I_ = 65.8 nM) that binds in a similar manner to **4d**, orienting its tail within this positively charged cleft (Figure 2B,F). Specifically, the phenolic -OH group is accommodated within the cleft, where it establishes an H-bond with the backbone of Pro193, suggesting that electrostatic complementarity between the target and the ligand is a key pharmacophoric requirement for strong inhibition.

Based on this hypothesis, the lack of interaction with the positively charged surface formed by Lys22, Trp23, and His84 is expected to weaken the inhibitory activity. Indeed, the **4a** isomer **4b** (*K*_I_ = 120nM) is not able to orient the phenolic group in the positively charged cleft due to the different positioning of the substituents in the aromatic ring (Figure 2C,G) and it does not interact with the Pro193 backbone. The predicted binding mode of **4g**, i.e., one of the weakest inhibitors of HpαCA of the **4a**–**j** series (*K*_I_ = 142 nM), further corroborates this structural hypothesis, showing that the phenol ring is prevented from approaching the positively charged cleft due to steric hindrance of the *ortho*-methyl groups (Figure 2D,H).

Similar conclusions were drawn from an analysis of docking poses against HpβCA, as the head portion binds similarly in all compounds, whereas changes in the orientation of the tail moieties were observed. Besides binding to the catalytic Zn(II) ion, the head of the compounds establishes H-bond interactions with Gln252 and Tyr280, as observed in the crystallographic structure of **AAZ** in complex with the homologue β-CA from *Coccomyxa* sp. [29] (residue numbering is in agreement with the sequence of HpβCA). The phenyl ring linked to the sulfonamide moiety occupies a position that is highly comparable with the available structural data and forms a π-stacking interaction with the side chain of Tyr307.

**Figure 3:**
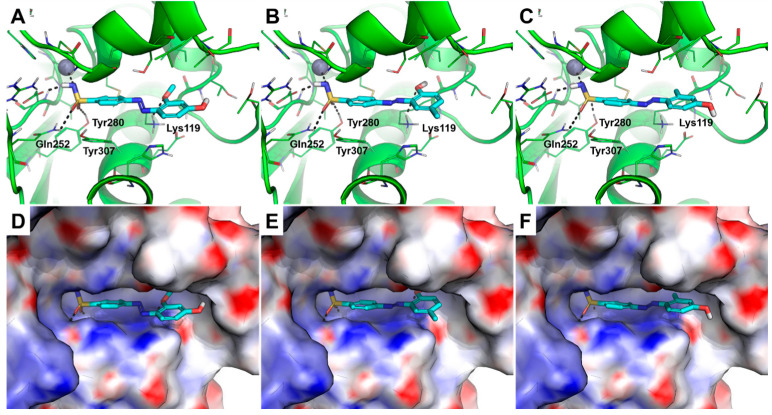
Predicted binding mode of compounds **4c** (**A**), **4h** (**B**), **4j** (**C**). The energy-minimized homology model of HpβCA is shown as a green cartoon and lines in panels (**A**–**C**), while the protein surface is colored according to the electrostatic surface potential in panels (**D**–**F**) (blue = positive charge, red = negative charge, white = uncharged lipophilic regions). Residues within 4 Å from the ligands are shown as green lines. Those of **4c**,**h**,**j** are shown as cyan sticks; non-polar hydrogen atoms are omitted. The catalytic Zn(II) ion is shown as a gray sphere. Polar interactions between inhibitors and HpβCA are highlighted by black dashed lines.

The strongest HpβCA inhibitor is **4c** (*K*_I_ = 17.1 nM), whose methoxyl group from the tail portion interacts with Lys119 through an H-bond. The phenolic group is projected towards the solvent area (Figure 3A,D).

Based on the molecular docking simulations, we hypothesized that the interaction with the Lys119 side chain and the exposure of a polar group to the solvent might be important features for potent HpβCA inhibition. In fact, two of the compounds that do not satisfy these structural requirements (i.e., **4h** and **4j**, *K*_I_ values of 127 and 138 nM, respectively) are among the weakest inhibitors of HpβCA of this series. In the case of **4h**, the phenolic -OH group interacts with Lys119 through an H-bond, but the molecule exposes a lipophilic methyl group to the solvent (Figure 3B,E), whereas **4j** fails to interact with Lys119 (Figure 3C,F).

Overall, the molecular modeling suggested that **4a**–**j** are able to bind in a highly similar manner to the α- and β-isoforms of the HpCA enzyme, at least in their head portion. Notably, subtle differences in the orientation of the tail portion within the variable regions of the enzyme nicely correlate with the experimental data and SARs, highlighting key interactions for the strong inhibition of HpαCA and HpβCA. The docking results are further corroborated by the remarkable correlation between the docking scores and experimental inhibition data (i.e., −log*K*_I_ values, Table S1). We did not perform the same molecular docking studies on series **5a**–**j,** because they were not tested against HpβCA. These structural hints could be easily translated to an additional series of primary sulfonamide-based HpCA inhibitors, driving the design of further derivatives equipped with alternative ZBGs.

### 2.4. Antimicrobial Activity versus H. pylori ATCC 43504

To further demonstrate the antimicrobial potential of these two series, all the compounds reported in Table 1 and Table 2 were tested in vitro against the reference strain *H. pylori* ATCC 43504 [30], and the Minimal Inhibitory (MIC) and Minimal Bactericidal (MBC) concentrations are reported in Table 3. This bacterium was selected based on the inhibitory potency evidenced by the two series of azobenzenesulfonamides against the HpCAs.

The lowest MIC value of 4 μg/mL was detected for two secondary azobenzenesulfonamides, **5c** and **5f**, while derivatives **4d** and **5h** showed the lowest antibacterial activity (MIC = 64 μg/mL). All the tested compounds proved to exert a bactericidal effect, showing an MBC/MIC ratio in the range of 1–2, and a strong impact of the substituents on the antibacterial effect was noticed. The best-in-class compounds were found to be **4c** and **4e** among the primary azobenzenesulfonamides **4a**–**j**, and **5c** and **5f** in the secondary azobenzenesulfonamide series (**5a**–**j**), showing themselves to exert a promising anti-*H. pylori* activity, with MIC values of 4–8 μg/mL and MBC values between 4 and 16 μg/mL. Compounds **5a**–**j** generally exhibited higher *K*_I_ values for bacterial CA compared to compounds **4a**–**j**. Particularly, in the cases of **5c** and **5f**, it was observed that the *K*_I_ values for bacterial CAs were higher than those of **4c** and **4e**. However, upon reviewing the results in Table 3, the MIC and MBC outcomes for **5c** and **5f** appear similar or even higher, thus suggesting different pharmacokinetic properties evidenced in the cell-based assays. This is also important, to avoid side effects involving interaction with human off-target CAs. The compounds of series **4** are potent inhibitors of HPCAs but they display a limited isoform selectivity over human ones, whereas the compounds of series **5** maintained a strong inhibition of bacterial CAs but were endowed with a minor interaction with the human isoforms. As expected, the MIC and MBC values of amoxicillin, used as the reference drug, are lower than the tested compounds, because CA is not essential in *H. pylori* but can hamper the bacterial performance, while amoxicillin targets an essential enzyme in the bacteria.

### 2.5. In Vivo Toxicity versus Galleria mellonella

Based on their antibacterial activity profile and aiming at forecasting a putative safety in vivo, **4c**,**e** and **5c**,**f** were selected for the evaluation of their acute and sub-acute toxicity on the *G. mellonella* in vivo model. In recent years, the use of *G. mellonella* (the greater wax moth) as an animal model, to study both bacterial infections and drug toxicity, has increased because of its simple usage, the possibility of performing large-scale studies, and the absence of ethical committee approval [32,33]. Despite the lack of an adaptive immune system, *G. mellonella* larvae have an innate system that shares similarities with that of mammals, allowing their use in preclinical studies [34,35]. *G. mellonella* is cultured and utilized worldwide in research laboratories as a model species for different entomological studies [36], as well as an alternative infection model to study diseases of humans and livestock [37,38]. One of the reasons for its wide use is that it can be reared in continuous colonies easily on a variety of diets. The larvae used in the present study were nurtured at the laboratory of entomology of the Department of Agricultural and Food Sciences of the University of Bologna, on a diet composed of milk powder, white and whole-wheat flour, maize flour, honey, bee wax, brewer’s yeast, and glycerin.

**Figure 4:**
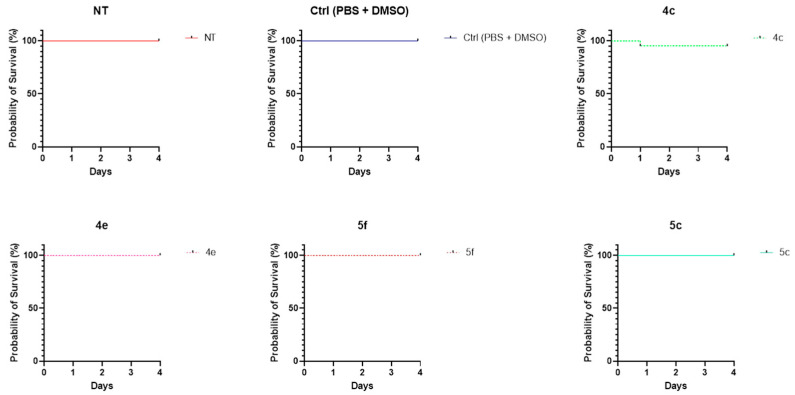
Effect of **4c** (80 μg/mL), **4e** (80 μg/mL), **5c** (40 μg/mL), and **5f** (40 μg/mL) on probability of *G. mellonella* survival (Kaplan–Meier survival curve [39]). NT is non-treated, Ctrl is control, only vehicle (PBS + DMSO).

*G. mellonella* sixth-instar larvae were injected with the tested compounds (at 10 × MIC values) or with vehicle control—Dulbecco’s Phosphate Buffer Saline (DPBS) and dimethylsulfoxide (DMSO). Only one larva died after 1 day of treatment with **4c**, showing similar survival rates as controls. As shown in Figure 4, there is no statistically significant difference between **4c** and controls, while for the other groups, the survival curves overlap with that of controls. These outcomes indicate no toxicity of **4c**,**e** and **5c**,**f** in *G. mellonella* larvae at the tested concentrations.

## 3. Conclusions

A new series of primary phenyldiazenyl sulfonamides and a previously published series of secondary azobenzene sulfonamides were evaluated as CA inhibitors by using a panel of human and bacterial CAs. All the primary sulfonamides **4a**–**j** were found to inhibit human CAs in the nanomolar range, while a more selective inhibitory trend could be noticed in data on bacterial isoenzymes. Furthermore, the secondary azobenzene sulfonamides **5a**–**j** showed a lower inhibitory potency compared to the primary ones. Considering that targeting HpCAs could be a good opportunity for the treatment of infections sustained by drug-resistant strains and that CA inhibitors are able to prevent *H. pylori* biofilm formation, these two series of azobenzene sulfonamides were also tested versus a reference strain of *H. pylori*. In particular, compounds **4c**,**e** and **5c**,**f** showed a promising anti-*H. pylori* activity, with MIC values of 4–8 μg/mL and MBC values between 4 and 16 μg/mL. These four compounds were also evaluated in vivo on the *G. mellonella* larva model and the results indicated no toxicity for **4c**,**e** and **5c**,**f** at the tested concentrations. Computational studies were also performed to predict the possible binding mode on both HpCAs of some azobenzenesulfonamides. The obtained results allowed the consideration of **4c**,**e** and **5c**,**f** as interesting starting points for the development of a new class of anti-*Helicobacter pylori* agents, taking into account the structural requirements suggested by in silico studies and the pharmacokinetic properties that can modulate cell-based antimicrobial activity [40]. Thus, new sulfonamide-containing antimicrobials could be the starting point to designing HpCA inhibitors with a high potency with respect to CA inhibitors presenting other moieties.

## 4. Materials and Methods

### 4.1. Chemistry, Synthesis, and Characterization of the New Azocompounds **4a**–**j**

A Varian instrument was utilized to record NMR spectra at 300 MHz using tetramethylsilane as an internal reference, and chemical shifts (*δ*) are reported in ppm. The splitting patterns are designated as s, singlet; d, doublet; t, triplet; q, quartet; dd, double doublet; m, multiplet; b, broad. Elemental analyses were carried out using a PerkinElmer 240 B microanalyzer and were found to be within ±0.4% of the theoretical values for C, H, and N for all compounds. The purity of all compounds was over 98%. All commercial and cell culture reagents, media, and reference compounds were obtained from Merk (Milan, Italy). Chemical reactions were monitored by thin-layer chromatography (TLC) on F254 silica gel 60 TLC plates. Flash chromatography was performed on silica gel 60 (Merck, Milan, Italy).

#### 4.1.1. General Procedure for Synthesis of Compounds **4a**–**j**

To a solution of sulfanilamide 1 (1 eq.) in HCl (1.5 mL, 6N) at 0 °C, we slowly added NaNO_2_ (1.5 eq.) in water at 0 °C to obtain the corresponding diazonium salt. After 1 h at 0 °C, this solution was mixed slowly to a proper phenol **3a**–**j** (1 eq.) dissolved in NaOH (2 mL, 4N) at 0 °C, letting it react for 4 h. After completion of the reaction, the pH was reduced at 2, adding 3N HCl. The formed precipitate was filtered under vacuum to obtain the desired products **4a**–**j,** used without further purification.

##### (*E*)-4-((2-hydroxy-5-methoxyphenyl)diazenyl)benzenesulfonamide **4a**

Brow solid, yield: 49%. ^1^H NMR (CD_3_OD) δ 3.83 (s, 3H, OC*H*_3_), 6.96–7.09 (dd, 2H, C*H*arom), 7.40 (d, 1H, C*H*arom), 8.07 (s, 4H, C*H*arom); ^13^C NMR (CD_3_OD) δ 54.9, 109.0, 118.6, 122.4, 122.6, 127.1, 137.7, 145.2, 148.7, 153.2, 153.3. Anal. calcd for C_13_H_13_N_3_O_4_S: C 50.81, H 4.26, N 13.67. Found C 50.98, H 4.25, N 13.65.

##### (*E*)-4-((4-hydroxy-3-methoxyphenyl)diazenyl)benzenesulfonamide **4b**

Orange solid, yield: 75%. ^1^H NMR (CD_3_OD) δ 3.94 (s, 3H, OC*H*_3_), 6.96 (d, 1H, C*H*arom), 7.55–7.60 (m, 2H, C*H*arom), 7.94–8.05 (dd, 4H, C*H*arom); ^13^C NMR (CD_3_OD) δ 54.9, 101.8, 110.0, 114.7, 122.2, 126.9, 146.1, 148.4, 153.5, 155.9, 160.0. Anal. calcd for C_13_H_13_N_3_O_4_S: C 50.81, H 4.26, N 13.67. Found C 50.85, H 4.27, N 13.68.

##### (*E*)-4-((4-hydroxy-2-methoxyphenyl)diazenyl)benzenesulfonamide **4c**

Orange solid, yield: 88%. ^1^H NMR (CD_3_OD) δ 3.97 (s, 3H, OC*H*_3_), 6.43–6.46 (m, 1H, C*H*arom), 6.59 (s, 1H, C*H*arom), 7.69 (d, 1H, C*H*arom), 7.92–8.02 (dd, 4H, C*H*arom); ^13^C NMR (CD_3_OD) δ 55.2, 99.4, 108.2, 110.0, 116.1, 121.4, 122.1, 126.8, 127.1, 143.8, 160.0. Anal. calcd for C_13_H_13_N_3_O_4_S: C 50.81, H 4.26, N 13.67. Found C 50.99, H 4.27, N 13.69.

##### (*E*)-4-((4-hydroxy-3-(trifluoromethyl)phenyl)diazenyl)benzenesulfonamide **4d**

Orange solid, yield: 49%. ^1^H NMR (CD_3_OD) δ 7.11 (d, 1H, C*H*arom), 7.94–8.09 (m, 5H, C*H*arom), 8.16 (d, 1H, C*H*arom); ^13^C NMR (CD_3_OD) δ 117.1, 122.1, 122.4, 122.6, 126.7, 126.9, 127.8, 144.7, 145.0, 154.2, 159.4. Anal. calcd for C_13_H_10_F_3_N_3_O_3_S: C 45.22, H 2.92, N 12.17. Found C 45.21, H 2.91, N 12.16.

##### (*E*)-4-((4-hydroxy-2-(trifluoromethyl)phenyl)diazenyl)benzenesulfonamide **4e**

Orange solid, yield: 53%. ^1^H NMR (CD_3_OD) δ 7.07 (dd, 1H, C*H*arom), 7.23 (d, 1H, C*H*arom), 7.89–7.93 (m, 1H, C*H*arom), 7.97–8.07 (dd, 4H, C*H*arom); ^13^C NMR (CD_3_OD) δ 113.0, 117.7, 119.1, 122.7, 122.8, 126.9, 130.9, 141.5, 145.1, 154.4, 162.3. Anal. calcd for C_13_H_10_F_3_N_3_O_3_S: C 45.22, H 2.92, N 12.17. Found C 45.23, H 2.93, N 12.18.

##### (*E*)-4-((4-hydroxy-2,5-dimethylphenyl)diazenyl)benzenesulfonamide **4f**

Orange solid, yield: 81%. ^1^H NMR (CD_3_OD) δ 2.17 (s, 3H, C*H*_3_), 2.62 (s, 3H, C*H*_3_), 6.72 (s, 1H, C*H*arom), 7.56 (s, 1H, C*H*arom), 7.89–8.03 (dd, 4H, C*H*arom); ^13^C NMR (CD3OD) δ 14.5, 15.9, 115.8, 117.5, 122.2, 123.3, 126.8, 139.8, 143.7, 143.9, 154.9, 160.1. Anal. calcd for C_14_H_15_N_3_O_3_S: C 55.07, H 4.95, N 13.76. Found 55.05, H 4.96, N 13.75.

##### (*E*)-4-((4-hydroxy-2,6-dimethylphenyl)diazenyl)benzenesulfonamide **4g**

Orange solid, yield: 79%. ^1^H NMR (CD_3_OD) δ 2.50 (s, 6H, aromC*H*_3_, aromC*H*_3_), 6.59 (s, 2H, C*H*arom), 7.89 (d, 2H, C*H*arom), 8.02 (d, 2H, C*H*arom); ^13^C NMR (CD_3_OD) δ 19.7, 115.8, 121.9, 126.9, 136.7, 142.8, 144.1, 155.2, 159.3. Anal. calcd for C_14_H_15_N_3_O_3_S: C 55.07, H 4.95, N 13.76. Found 55.10, H 4.94, N 13.77.

##### (*E*)-4-((2-hydroxy-5-methylphenyl)diazenyl)benzenesulfonamide **4h**

Orange solid, yield: 47%. ^1^H NMR (Acetone) δ 2.38 (s, 3H, C*H*_3_), 6.81 (s, 2H, NH_2_), 6.96 (d, 1H, C*H*arom), 7.32 (d, 1H, C*H*arom), 7.79 (s, 1H, C*H*arom), 8.08–8.16 (dd, 4H, C*H*arom), 11.85 (s, 1H, OH); ^13^C NMR (Acetone) δ 19.3, 117.9, 122.6, 127.4, 129.5, 130.7, 135.5, 137.4, 145.9, 151.2, 152.8. Anal. calcd for C_13_H_13_N_3_O_3_S: C 53.60, H 4.50, N 14.42. Found 53.48, H 4.51, N 14.45.

##### (*E*)-4-((4-hydroxy-3-methylphenyl)diazenyl)benzenesulfonamide **4i**

Yellow solid, yield: 79%. ^1^H NMR (CD_3_OD) δ 2.27 (s, 3H, C*H*_3_), 4.65 (s, 2H, NH_2_), 6.89 (d, 1H, C*H*arom), 7.68–7.75 (m, 2H, C*H*arom), 7.93 (d, 2H, C*H*arom), 8.02 (d, 2H, C*H*arom); ^13^C NMR (CD_3_OD) δ 14.9, 114.3, 122.2, 123.5, 125.1, 125.4, 126.9, 144.2, 145.9, 154.6, 160.0. Anal. calcd for C_13_H_13_N3O_3_S: C 53.60, H 4.50, N 14.42. Found 53.51, H 4.49, N 14.43.

##### (*E*)-4-((4-hydroxy-2-methylphenyl)diazenyl)benzenesulfonamide **4j**

Red solid, yield: 81%. ^1^H NMR (CD_3_OD) δ 2.67 (s, 3H, C*H*_3_), 6.68 (d, 1H, C*H*arom), 6.77 (s, 1H, C*H*arom), 7.68 (d, 1H, C*H*arom), 7.92–7.95 (m, 2H, C*H*arom), 8.02 (d, 2H, C*H*arom); ^13^C NMR (CD_3_OD) δ 16.4, 110.0, 113.6, 116.7, 116.8, 122.3, 126.9, 142.2, 144.0, 154.9, 161.6. Anal. calcd for C_13_H_13_N_3_O_3_S: C 53.60, H 4.50, N 14.42. Found 53.59, H 4.49, N 14.44.

### 4.2. Carbonic Anhydrases Inhibition Assay

An Applied Photophysics stopped-flow instrument was used to evaluate the ability of the test compounds to inhibit CA-catalyzed CO_2_ hydration [41]. An amount of 0.2 mM Phenol red was chosen as an indicator (absorbance maximum = 557 nm), with 20 mM HEPES (pH 7.4 for α- and γ-CAs, 8.4 for β-CAs) as a buffer, and 20 mM Na_2_SO_4_ (to maintain a constant ionic strength), following the initial rates of the CA-catalyzed CO_2_ hydration reaction for a period of 10–100 s. The CO_2_ concentrations ranged from 1.7 to 17 mM for the determination of the kinetic parameters and inhibition constants. Enzyme concentrations spanned from 5 to 12 nM. Stock solutions of the inhibitor (0.1 mM) were prepared in distilled–deionized water, and dilutions up to 0.01 nM were prepared with the assay buffer. Inhibitor and enzyme solutions were preincubated for 15 min at r.t. for the formation of the E–I complex. The inhibition constants were obtained by non-linear least-squares methods using PRISM 3 and the Cheng–Prusoff equation and represent the mean from at least three different determinations. Apart from human CAs I and II purchased from Merck, all the bacterial CAs were recombinant and obtained in-house [42].

### 4.3. Molecular Modeling

Ligands were sketched in 2D with Picto software (version 4.4.0.4. OpenEye Scientific Software, Santa Fe, NM, USA) [43] and converted into 3D structures with the OMEGA2 program (version 3.1.0.3. OpenEye Scientific Software, Santa Fe, NM, USA) [44]. Ionization of molecules was carried out by QUACPAC (version 2.0.0.3. OpenEye Scientific Software, Santa Fe, NM, USA) [45] for a pH of 7.4, whereas the sulfonamide group was considered as deprotonated. It is worth mentioning that compounds bearing a phenolic group were docked either in a protonated or non-protonated form (phenolate ions), with no differences in the binding mode. For the sake of clarity, the neutral phenolic form is shown in the figures.

Ligand energy minimization was performed with SZYBKI (version 1.10.0.3. OpenEye Scientific Software, Santa Fe, NM, USA) [46].

The homology model of HpβCA was generated using the Prime software (version 5.5) [47] included in the Maestro suite (version 11.9.011) [48], using the HpβCA sequence (UniProt ID: Q9ZN54, strain J99) as the target sequence and the crystallographic structure of the βCA from the *Coccomyxa* sp. (PDB ID: 3UCJ) [29] as a structural template. The AAZ molecule that coordinates the catalytic Zn(II) ion in the structural template was kept in place during the homology modeling, to guarantee the generation of an enzyme structure with the catalytic site in the open/inhibited form. Energy minimization of the HpβCA homology model was carried out with Amber18 [49]. The ff14SB force field was used for the protein, while GAFF was used for **AAZ**. The catalytic Zn(II) ion was assigned an arbitrary charge of +1 according to previous works [50], and its coordination was parametrized with a non-bonded approach. The rough homology model was solvated in a box of TIP3P-type water molecules buffering 8 Å from the macromolecules, while Na^+^ ions were added to achieve charge neutrality. The solvent was first energy-minimized for 500 steps using the steepest descent algorithm (SD), followed by 1500 steps with the conjugate gradient algorithm (CG) while keeping the solute frozen. Then, the solvated solute was energy-minimized for 1000 steps with the SD and a subsequent 6500 steps with the CG.

The crystallographic structure of the HpαCA, coded by PDB ID 4XFW [29], and the energy-minimized homology model of HpβCA, were used as rigid receptors in molecular docking simulations performed with the GOLD program (version 2020-1) [51]. The catalytic Zn(II) ion was selected as the center of the binding site, having an amplitude of 10 Å. The metal ion was treated as a tetracoordinate heteroatom. For each ligand, 10 runs of the Genetic Algorithm (GA) were performed. The CHEMPLP scoring function with default parameters was used, while the GA search efficiency was increased up to 200%.

### 4.4. Antibacterial Susceptibility Testing

*H. pylori* ATCC 43504 was cultured on a Columbia Agar base (Becton Dickinson and Company, Franklin Lakes, NJ, USA) supplemented with 10% horse serum (Merck, St. Louis, MO, USA) and 0.25% yeast extract (Oxoid Ltd., Hampshire, UK), incubated at 37 °C for 72 h in a microaerophilic atmosphere (CampyGen, Thermo Fisher Scientific, Milan, Italy). Colonies were collected and transferred into 10 mL of Brain Heart Infusion broth (BHI, Oxoid Ltd., Hampshire, UK) supplemented with 5% (*v*/*v*) of fetal bovine serum (FBS, Merck, Milan, Italy) and incubated overnight at 37 °C, at 125 rpm under microaerophilic conditions. After 18 h of incubation, the broth culture was diluted to obtain a bacterial suspension of 10^6^ CFU/mL. MICs were determined by three different assays: the broth micro-dilution method, the alamarBlue assay, and the CFU count.

The broth micro-dilution method was performed according to the Clinical & Laboratory Standards Institute guidelines [52]. Each compound was resuspended in DMSO, and serial two-fold dilutions were performed directly in 96-well plates to obtain a concentration range of 0.0625–128 µg/mL (the final percentage of DMSO was 0.57%). To obtain a final concentration of 10^5^ CFU/mL, 10 µL of the sample, containing 10^6^ CFU/mL, was added to each well, in which 90 µL of the tested compound had been previously placed. The controls consisted of (I) *H. pylori* broth culture in BHI plus 5% FBS; (II) *H. pylori* broth culture in BHI plus 5% FBS added with 0.57% DMSO; (III) only BHI plus 5% FBS; (IV) only BHI plus 5% FBS added with 0.57% DMSO. Three independent experiments were performed in triplicate. Amoxicillin, one of the clinically used drugs, was compared as a positive control. The plates were incubated at 37 °C for 72 h under microaerophilic conditions.

The alamarBlue assay (AB; Thermo Fisher Scientific, Waltham, MA, USA) was conducted as previously described [17]. The results were further confirmed by the CFU count, which was carried out from the wells stained with alamarBlue. Briefly, 100 µL from each well was resuspended in 900 µL of DPBS (Merck, St. Louis, MO, USA), and serial dilutions were then performed in DPBS, plated on agar, and incubated at 37 °C under microaerophilic conditions for 3–5 days. The MBC was defined as the lowest concentration of the compound that kills 99.9% of the test bacteria and was determined by CFU counts based on the spreading of 100 µL, taken from the wells after the MIC of the treated samples and controls on the selected agar.

### 4.5. Evaluation of In Vivo Toxicity on Galleria mellonella Model

*G. mellonella* larvae were stored in the dark at 20 °C. Larvae weighing 200–250 mg were used for the experiment. Three experimental groups consisting of (I) larvae treated with the chosen compound; (II) untreated larvae (not injected); and (III) larvae injected with PBS added with the same percentage of DMSO obtained in the treated group were evaluated. Each experimental group counted 10 larvae, and each larva was injected into the third left proleg using a Hamilton syringe (Hamilton, Reno, NV, USA). A total volume of 10 μL of the sample was administered to each animal. In particular, concentrations equal to the MIC of the administered compounds were increased 10-fold (10 × MIC) [53], suggesting that one should consider in the *G. mellonella* in vivo model a dilution factor corresponding to 10. Each compound was diluted in PBS, starting from their DMSO stock solutions. Finally, the larvae were housed in Petri dishes at 37 °C and monitored daily for 4 days to score mortality (the larvae were considered dead when they were unresponsive to touch and were unable to correct themselves when rolled onto their back) [54]. Two independent experiments were performed.

## Figures and Tables

**Table 1:** Inhibition data of primary azobenzenesulfonamides **4a**–**j** and reference compound **AAZ** on panel of human and bacterial CAs.

CPD		*K*_I_ (nM) *											
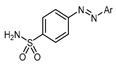		Human Isoforms				Bacterial Isoforms							
		hCA I	hCA II	hCA IX	hCA XII	Pgi βCA	Pgi γCA	Hp αCA	Hp βCA	Eco βCA	Ps CA3	Msc CA	Smu CA
N	Ar												
**4a**	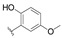	45	24.1	201	9.5	803	3438	65.8	74.1	400	228	2931	503
**4b**	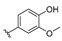	34.8	3.3	831	21.8	1921	837	120	49.7	95.8	702	2811	649
**4c**	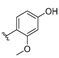	84.9	5.4	545	34.7	3628	2004	70.9	17.1	96.3	221	7764	289
**4d**	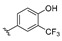	20.1	4.8	182	40.2	3167	94.7	39	19.4	738	222	6423	444
**4e**	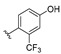	5.4	4.2	191	54.1	899	347	89.2	68.8	95.3	495	5720	213
**4f**	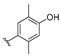	5.7	1.9	97.1	56.7	3760	472	69.4	70.6	867	191	5694	215
**4g**	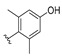	4.9	3.7	171	63.9	3668	939	142	74.3	550	879	7282	500
**4h**	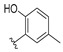	68.4	6.6	184	9.6	3850	772	130	127	360	980	5316	82.1
**4i**	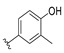	4.4	3.4	181	7.5	2071	6326	140	119	2115	114	7468	368
**4j**	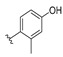	5.3	4.7	213	84.1	5778	914	72.2	138	3044	1845	6316	296

**AAZ** 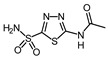		250	12.1	25.8	5.7	214	324	21.4	40	227	75.9	628	344

* Mean from three different assays by a stopped-flow technique (errors were in the range of ±5–10% of the reported values). **AAZ** was used as a reference control. Pgi: *Porphyromonas gingivalis*; Hp: *Helicobacter pylori*; Eco: *Escherichia coli*; Ps: *Pseudomonas aeruginosa*; Msc: *Mammaliiccous (Staphylococcus) sciuri*; Smu: *Streptococcus mutans*.

**Table 2:** Inhibition data of secondary azobenzenesulfonamides **5a**–**j** and reference compound **AAZ** on panel of human and bacterial CAs.

CPD		*K*_I_ (nM)									
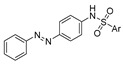		Human Isoforms				Bacterial Isoforms					
		hCA I	hCA II	hCA IX	hCA XII	Pgi βCA	Pgi γCA	Hp αCA	Eco βCA	Ps CA3	Msc CA
	Ar										
**5a**	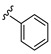	209	n.a.	3977	955	n.a.	n.a.	460	6949	9284	22,845
**5b**	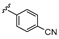	359	n.a.	614	760	71,449	n.a.	75.6	73,361	5760	6962
**5c**	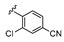	908	24,679	9196	1750	n.a.	n.a.	77.6	9206	n.a.	54,752
**5d**	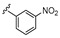	6749	n.a.	601	215	n.a.	n.a.	70.9	9081	90178	n.a.
**5e**	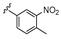	853	n.a.	792	847	n.a.	n.a.	604	58,996	n.a.	8595
**5f**	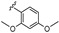	905	n.a.	5739	894	n.a.	n.a.	71.3	77,702	n.a.	n.a.
**5g**	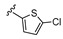	905	n.a.	4043	4117	n.a.	n.a.	55.8	59,165	n.a.	28,418
**5h**	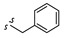	5531	n.a.	4495	2254	n.a.	n.a.	79	88,538	n.a.	7515
**5i**	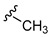	5990	970,324	599	2240	n.a.	n.a.	89.8	6951	9284	n.a.
**5j**	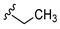	931	n.a.	7075	3335	n.a.	n.a.	68.4	n.a.	69,829	28,728

**AAZ**		250	12.1	25.8	5.7	214	324	21.4	227	75.9	344

Mean from 3 different assays by a stopped-flow technique (errors were in the range of ±5–10% of the reported values). **AAZ** was used as a reference control. n.a. = not active at the highest concentration tested (100 µM).

**Table 3:** MIC and MBC values of the tested compounds (**4a**–**j** and **5a**–**j**) and amoxicillin as the reference drug (μg/mL) against *H. pylori* ATCC 43504.

CPD	MIC * (μg/mL)	MBC * (μg/mL)
**4a**	8	32
**4b**	32	32
**4c**	8	16
**4d**	64	128
**4e**	8	16
**4f**	32	32
**4g**	16	32
**4h**	32	64
**4i**	32	64
**4j**	16	32
**5a**	16	32
**5b**	32	64
**5c**	4	8
**5d**	16	64
**5e**	8	16
**5f**	4	4
**5g**	16	16
**5h**	64	128
**5i**	16	32
**5j**	16	16
Amoxicillin	0.064	0.064

* MIC values are reported as the means of three independent experiments via the broth micro-dilution method, the alamarBlue assay, and the colony-forming unit (CFU) count, while MBC values are via the CFU count [31]. Amoxicillin was used as a reference antibiotic in these assays.

## Data Availability

The original contributions presented in the study are included in the article/Supplementary Material, further inquiries can be directed to the corresponding authors.

## Supplementary Materials

The following supporting information can be downloaded at: https://www.mdpi.com/article/10.3390/ph17081027/s1. Table S1: Docking scores of compounds **4a**–**j** to HpαCA and HpβCA isoforms. ^1^H and ^13^C spectra of final compounds **4a**–**j**.
