# Supplementary material for: Azobenzenesulfonamide Carbonic Anhydrase Inhibitors as New Weapons to Fight Helicobacter pylori: Synthesis, Bioactivity Evaluation, In Vivo Toxicity, and Computational Studies
Source: Pharmaceuticals (Basel). 2024 Aug 5;17(8):1027. doi: 10.3390/ph17081027 (PMC11357054; doi:10.3390/ph17081027)
Supplement: Supplementary file 1 [file pharmaceuticals-17-01027-s001.zip › pharmaceuticals-3109758-supplementary.pdf]

Supplementary Materials

Azobenzenesulfonamide Carbonic Anhydrase Inhibitors as New Weapons to Fight *Helicobacter pylori*: Synthesis, Bioactivity Evaluation, In Vivo Toxicity, and Computational Studies

Letizia Giampietro [1,*,†], Beatrice Marinacci [1,2,†], Alice Della Valle [1], Ilaria D'Agostino [3], Aldo Lauro [4], Mattia Mori [4], Simone Carradori [1], Alessandra Ammazzalorso [1], Barbara De Filippis [1], Cristina Maccallini [1], Andrea Angeli [5], Clemente Capasso [6], Santolo Francati [7], Adriano Mollica [1], Rossella Grande [1,8] and Claudiu T. Supuran [5]

[1] Department of Pharmacy, "G. d'Annunzio" University of Chieti-Pescara, 66100 Chieti, Italy
[2] Department of Innovative Technologies in Medicine & Dentistry, "G. d'Annunzio" University of Chieti-Pescara, 66100 Chieti, Italy
[3] Department of Pharmacy, University of Pisa, Via Bonanno 6, 56126 Pisa, Italy
[4] Department of Biotechnology, Chemistry and Pharmacy, University of Siena, Via Aldo Moro 2, 53100 Siena, Italy
[5] Neurofarba Department, University of Florence, Sesto Fiorentino, 50019 Florence, Italy
[6] Department of Biology, Agriculture and Food Sciences, National Research Council (CNR), Institute of Biosciences and Bioresources, 80131 Naples, Italy
[7] Department of Agricultural and Food Sciences (DISTAL), University of Bologna, 40126 Bologna, Italy
[8] Center for Advanced Studies and Technology, "G. d'Annunzio" University of Chieti-Pescara, 66100 Chieti, Italy
* Correspondence: letizia.giampietro@unich.it
† These authors contributed equally to this work.

Contents:

Figure S1. ^{1}H and ^{13}C spectra of final compounds 4a-j.

Table S1. Docking scores of compounds 4a-j to HpCAα and HpCAβ isoforms.

Figure S1.

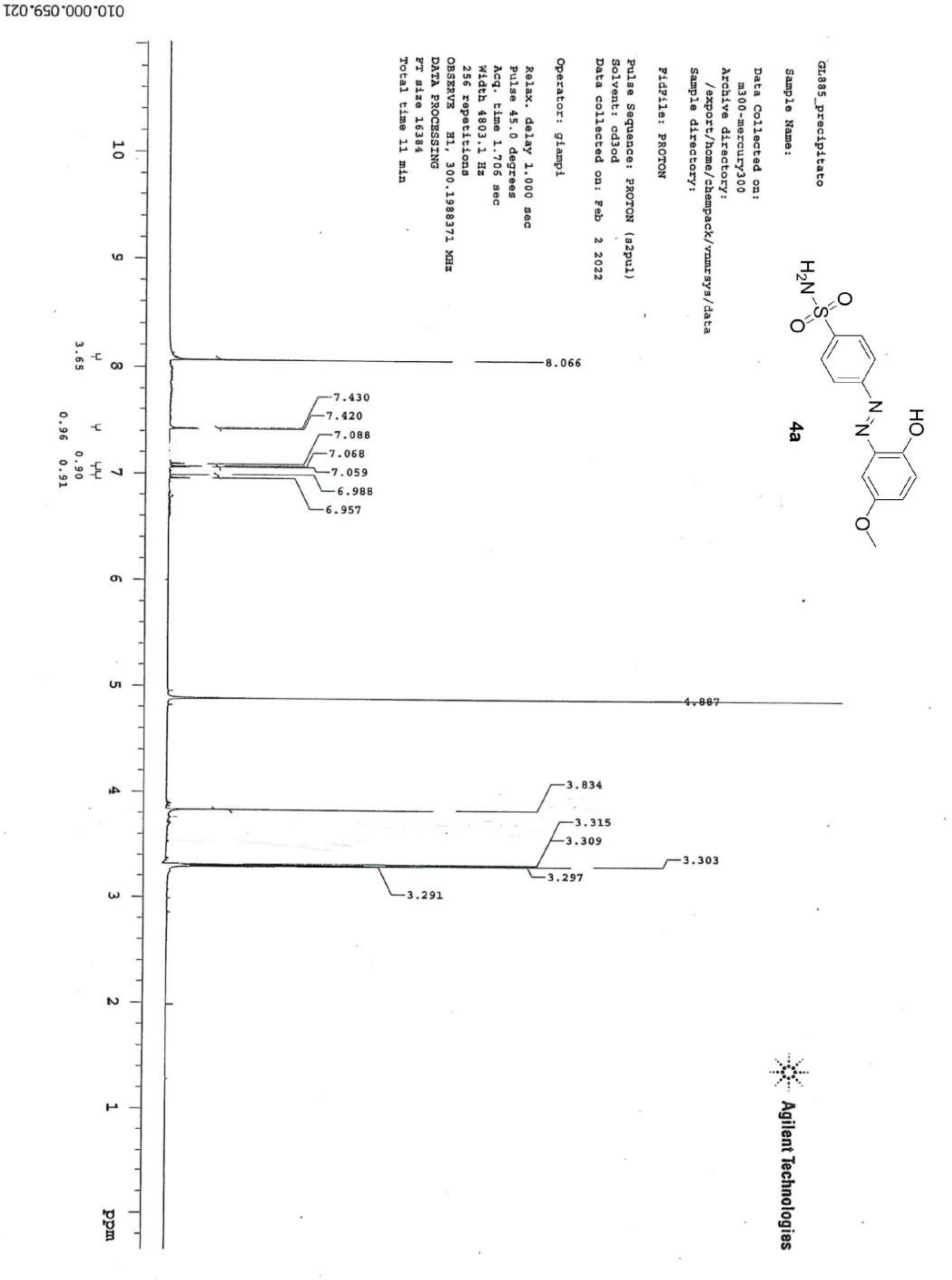
GL885_precipitato
Sample Name:
Data Collected on:
m300-mercury300
Archive directory:
/export/home/chempack/vnmrsys/data
Sample directory:
FidFile: PROTON
Pulse Sequence: PROTON (s2pul)
Solvent: cd3od
Data collected on: Feb 2 2022
Operator: giampi
Relax. delay 1.000 sec
Pulse 45.0 degrees
Acq. time 1.706 sec
Width 4803.1 Hz
256 repetitions
OBSERVE H1, 300.1988371 MHz
DATA PROCESSING
FT size 16384
Total time 11 min
4a
8.066
7.430
7.420
7.088
7.068
7.059
6.988
6.957
4.887
3.834
3.315
3.309
3.303
3.297
3.291
3.65
0.96
0.90
0.91
ppm
Agilent Technologies

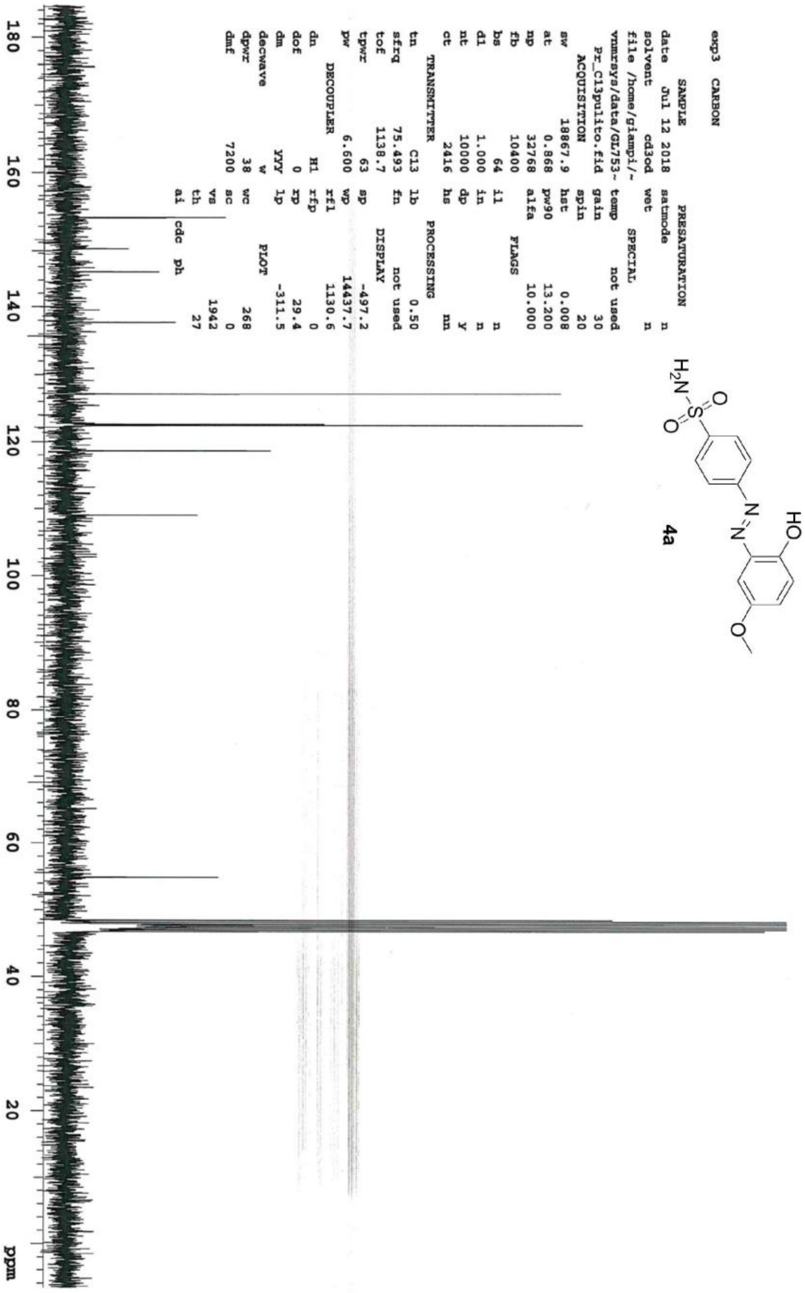
exp3 CARBON
SAMPLE
PRESATURATION
date Jul 12 2018 satmode n
solvent cd3od wet n
file /home/giampi/~ SPECIAL
vnmrsys/data/GL753~ temp not used
Pr_C13pulito.fid gain 30
ACQUISITION spin 20
sw 18867.9 hst 0.008
at 0.868 pw90 13.200
np 32768 alfa 10.000
fb 10400 FLAGS
bs 64 il n
d1 1.000 in n
nt 10000 dp y
ct 2416 hs nn
TRANSMITTER PROCESSING
tn C13 lb 0.50
sfrq 75.493 fn not used
tof 1138.7 DISPLAY
tpwr 63 sp -497.2
pw 6.600 wp 14437.7
DECOUPLER rfl 1130.6
dn H1 rfp 0
dof 0 rp 29.4
dm yyy lp -311.5
decwave w PLOT
dpwr 38 wc 268
dmf 7200 sc 0
vs 1942
th 27
ai cdc ph
180
160
140
120
100
80
60
40
20
ppm
H2N
S
O
O
N
N
HO
O
4a

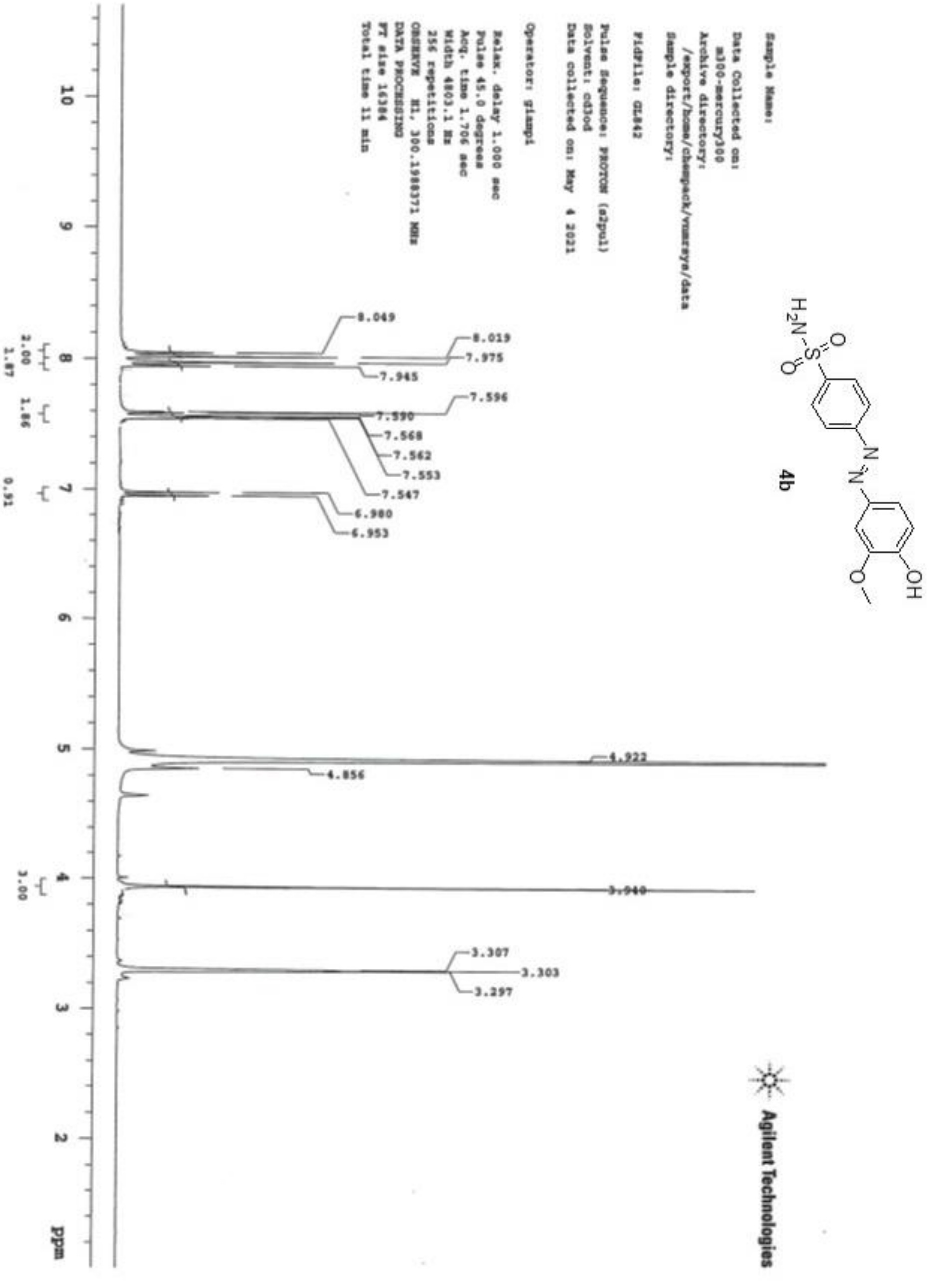
Sample Name:
Data Collected on:
m300-mercury300
Archive directory:
/export/home/chempack/vnmrsys/data
Sample directory:
FidFile: GL842
Pulse Sequence: PROTON (s2pul)
Solvent: cd3od
Data collected on: May 4 2021
Operator: giampi
Relax. delay 1.000 sec
Pulse 45.0 degrees
Acq. time 1.706 sec
Width 4803.1 Hz
256 repetitions
OBSERVE H1, 300.1988371 MHz
DATA PROCESSING
FT size 16384
Total time 11 min
4b
OH
8.049
8.019
7.975
7.945
7.596
7.590
7.568
7.562
7.553
7.547
6.980
6.953
4.922
4.856
3.940
3.307
3.303
3.297
2.00
1.87
1.86
0.91
3.00
ppm
Agilent Technologies

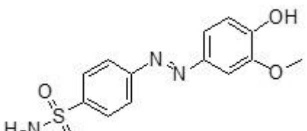

4b

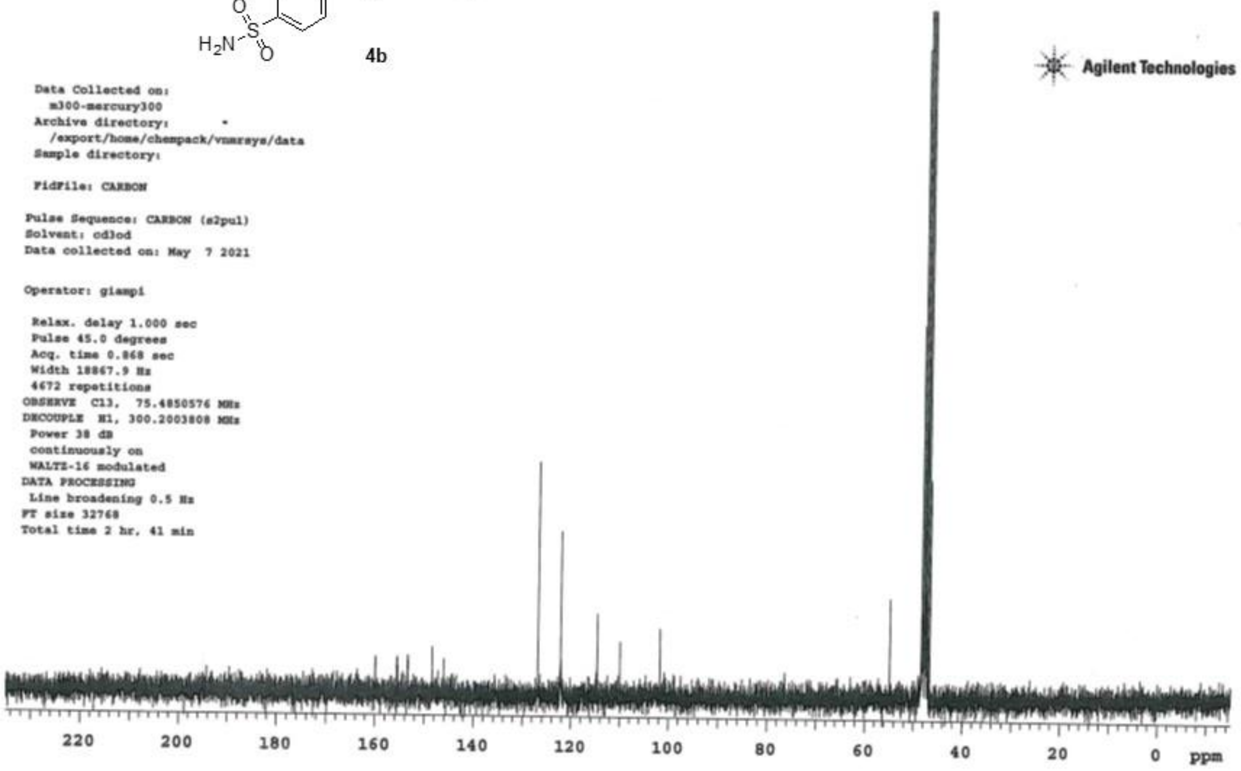
Data Collected on:
m300-mercury300
Archive directory:
/export/home/chempack/vnmrsys/data
Sample directory:
FidFile: CARBON
Pulse Sequence: CARBON (s2pul)
Solvent: cd3od
Data collected on: May 7 2021
Operator: giampi
Relax. delay 1.000 sec
Pulse 45.0 degrees
Acq. time 0.868 sec
Width 18867.9 Hz
4672 repetitions
OBSERVE C13, 75.4850576 MHz
DECOUPLE H1, 300.2003808 MHz
Power 38 dB
continuously on
WALTZ-16 modulated
DATA PROCESSING
Line broadening 0.5 Hz
FT size 32768
Total time 2 hr, 41 min
Agilent Technologies
220
200
180
160
140
120
100
80
60
40
20
0
ppm

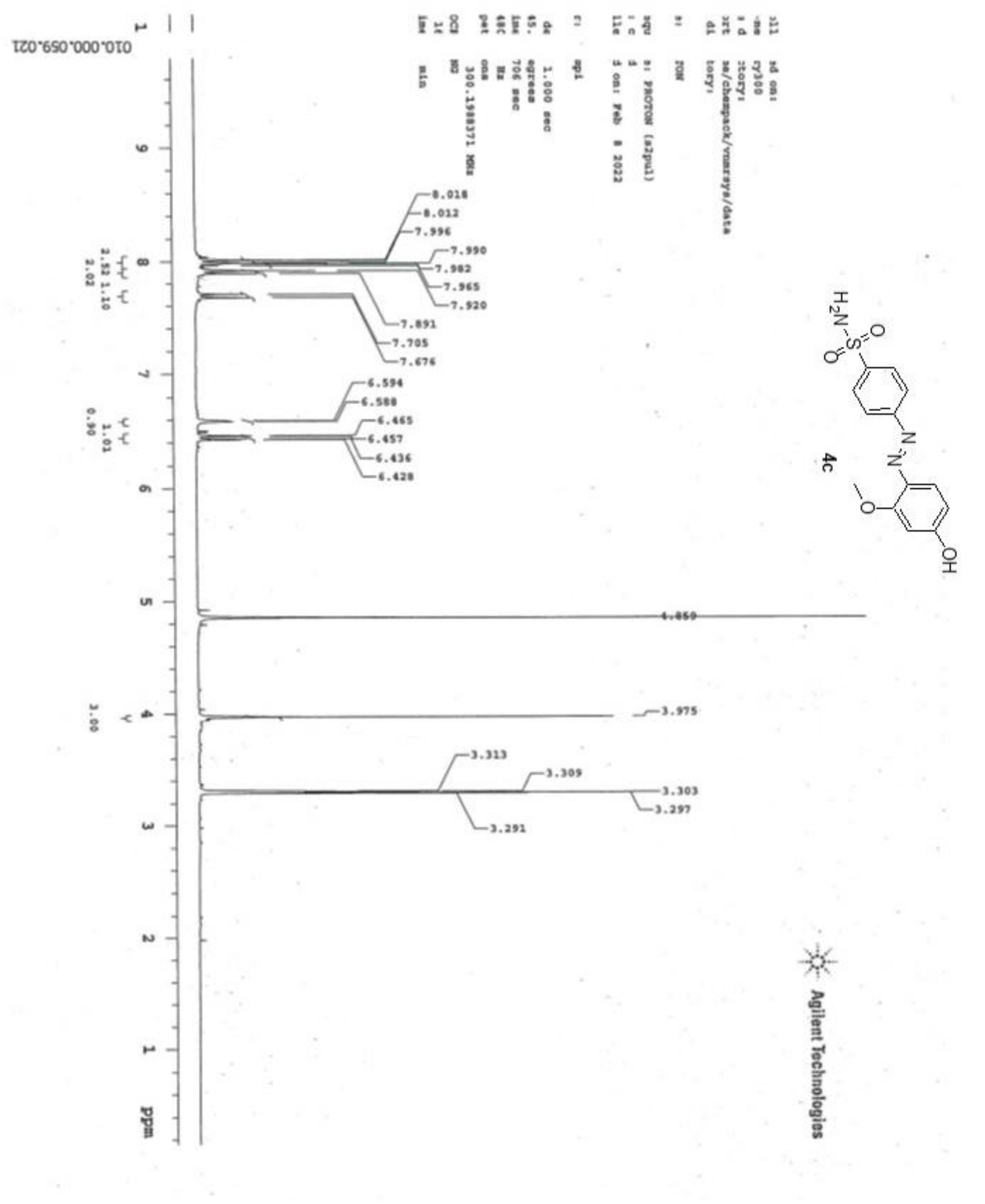

4c
Agilent Technologies
ppm

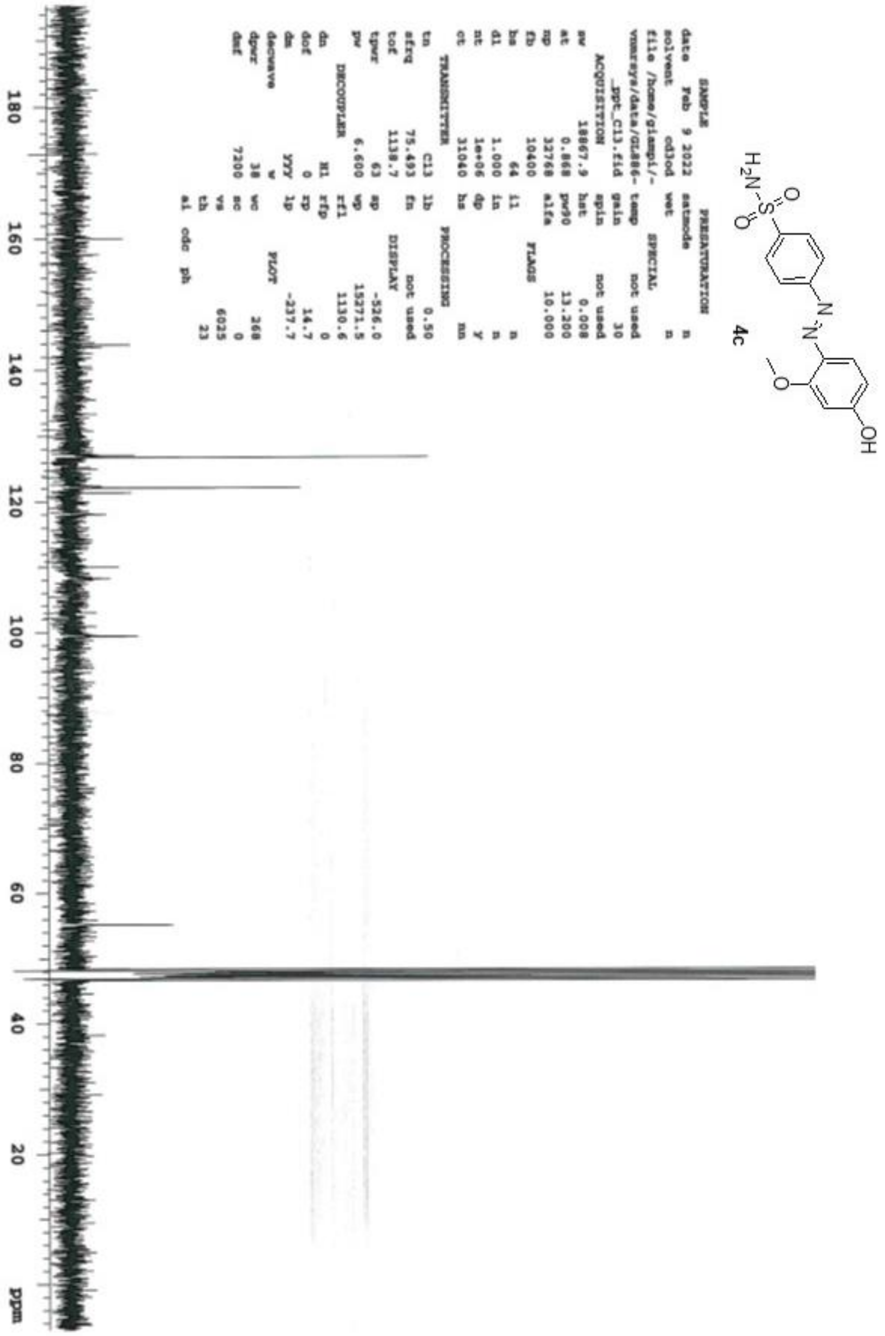

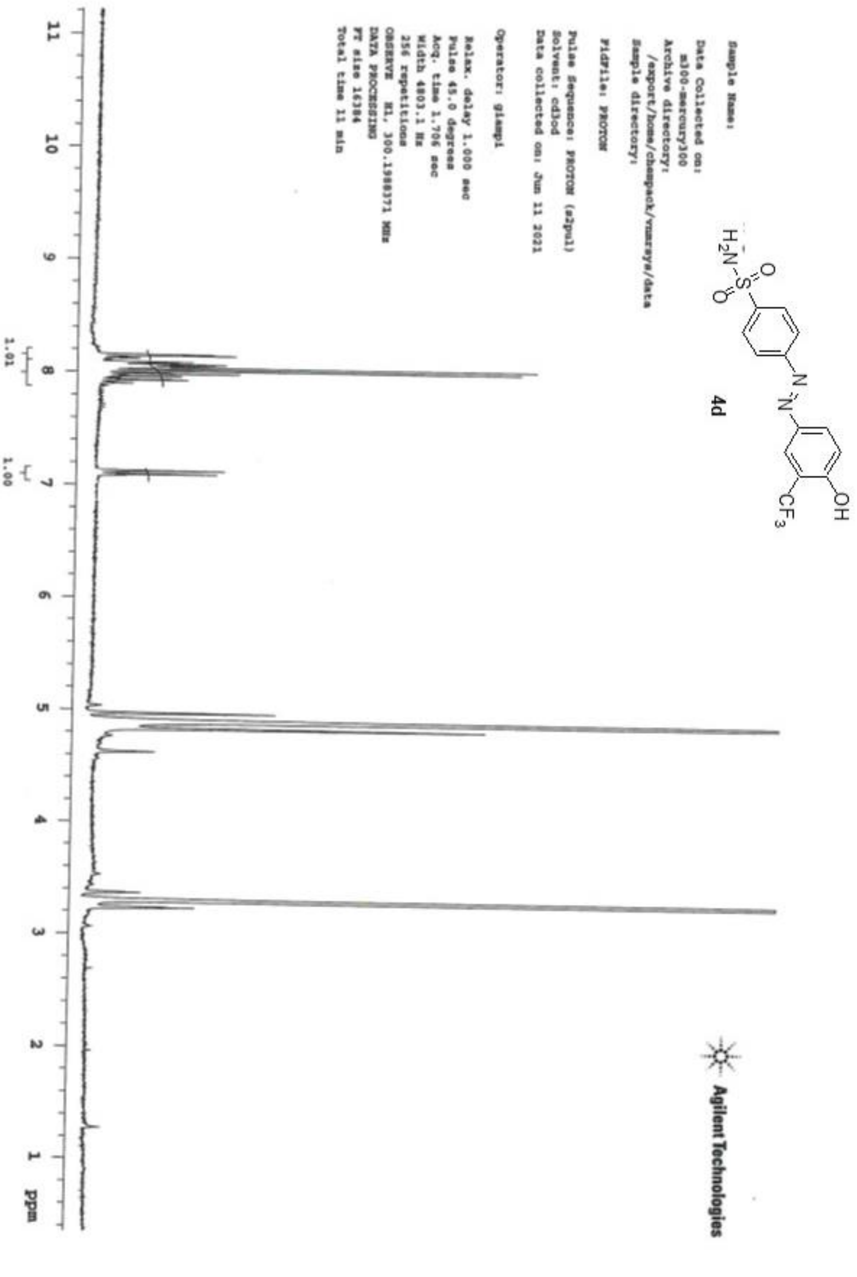
Sample Name:
Data Collected on:
s300-mercury300
Archive directory:
/export/home/chempack/vnmrsys/data
Sample directory:
FidFile: PROTON
Pulse Sequence: PROTON (s2pul)
Solvent: cd3od
Data collected on: Jun 11 2021
Operator: gismpi
Relax. delay 1.000 sec
Pulse 45.0 degrees
Acq. time 1.706 sec
Width 4803.1 Hz
256 repetitions
OBSERVE H1, 300.1988371 MHz
DATA PROCESSING
FT size 16384
Total time 11 min
4d
OH
CF_3
Agilent Technologies
ppm

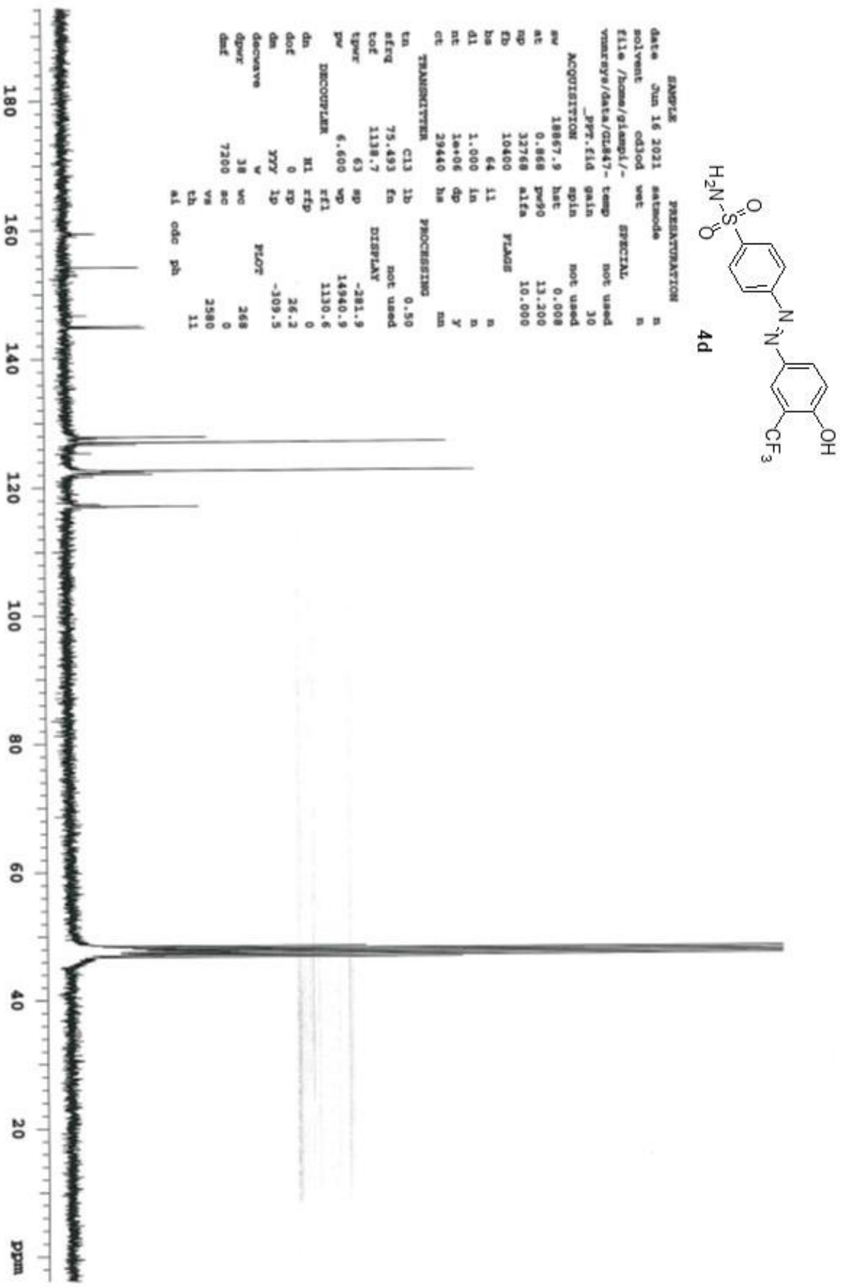
4d
OH
CF3
H2N
180
160
140
120
100
80
60
40
20
ppm

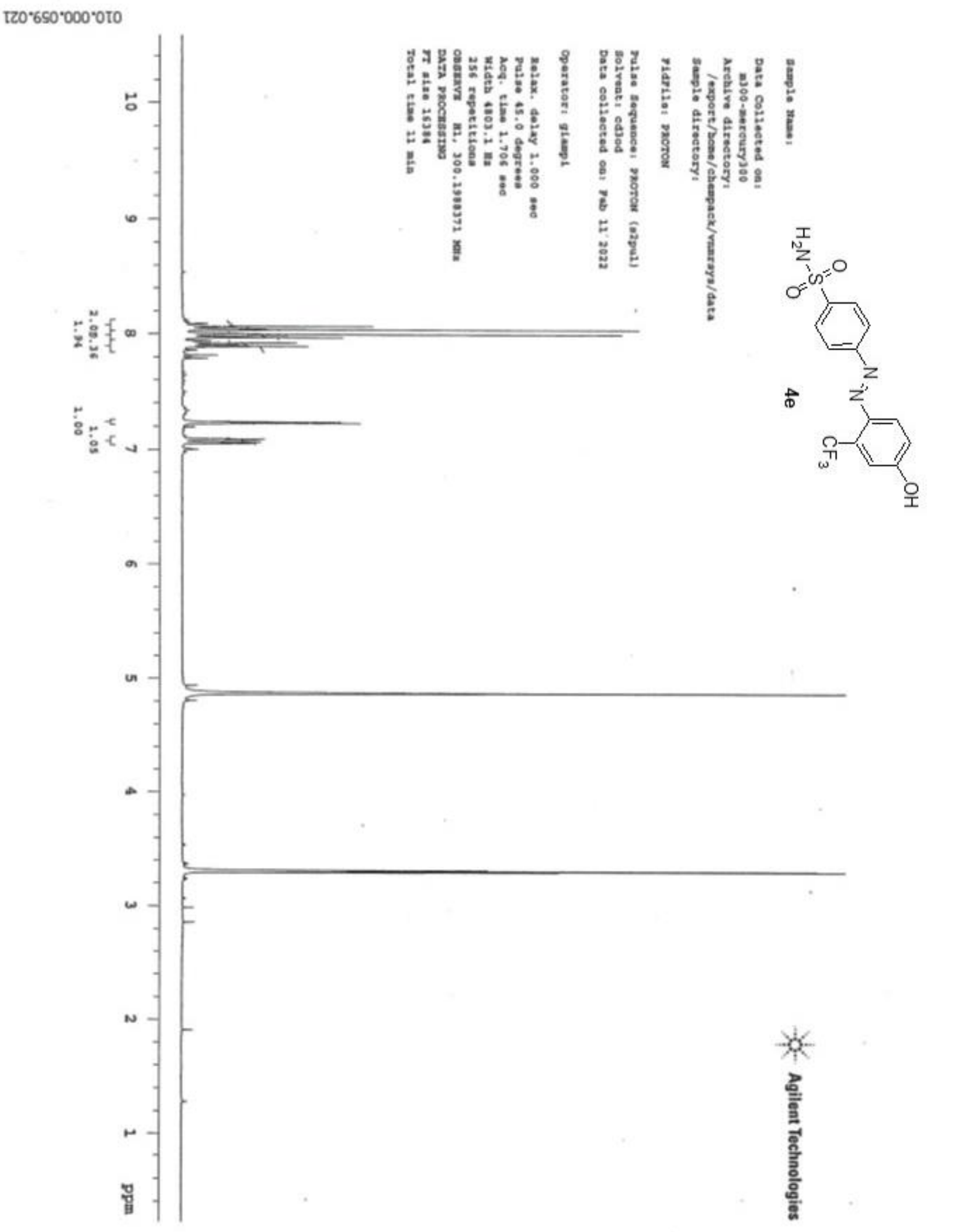
4e
Agilent Technologies
ppm

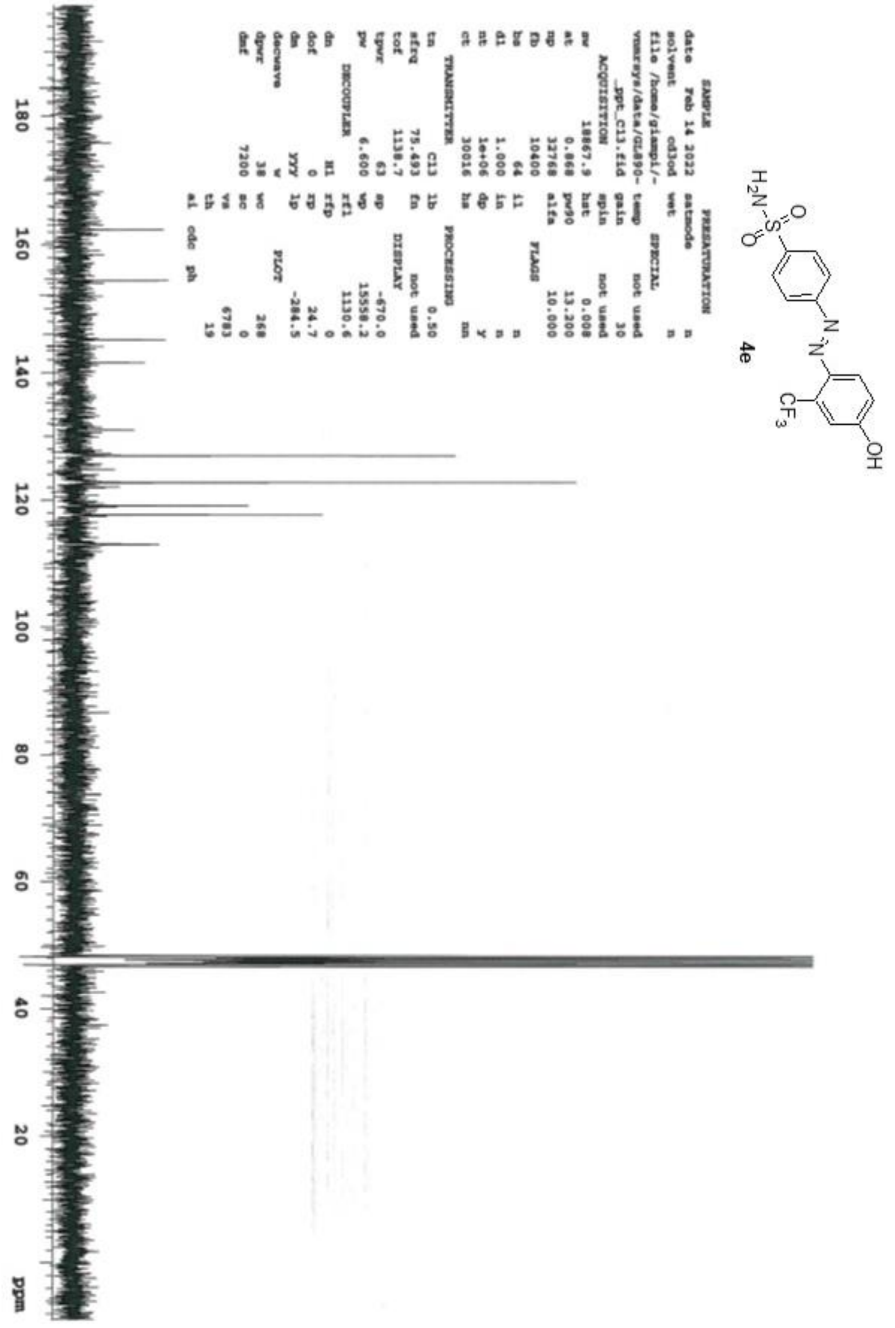
4e

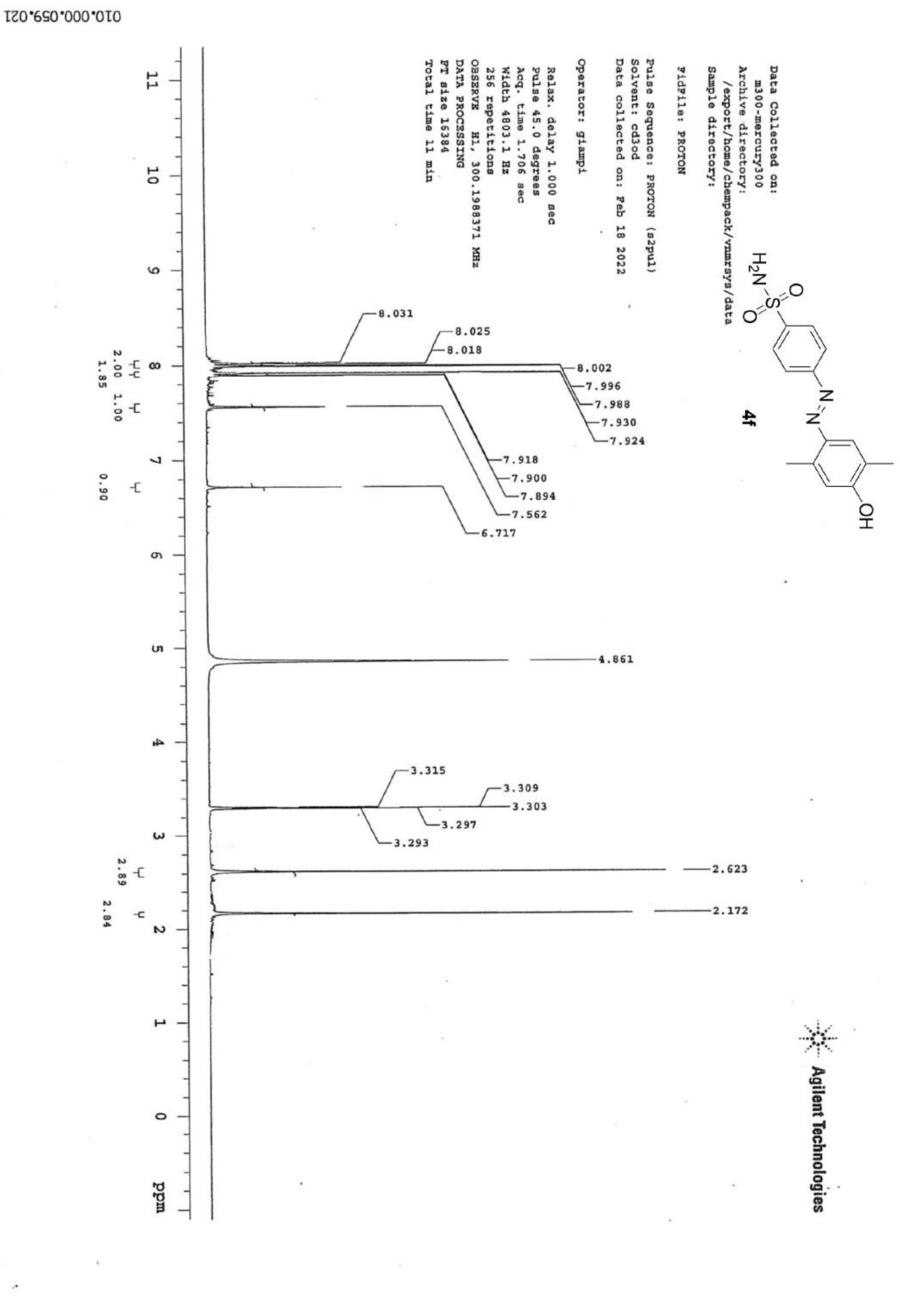
Data Collected on:
m300-mercury300
Archive directory:
/export/home/chempack/vnmrsys/data
Sample directory:
FidFile: PROTON
Pulse Sequence: PROTON (s2pul)
Solvent: cd3od
Data collected on: Feb 18 2022
Operator: giampi
Relax. delay 1.000 sec
Pulse 45.0 degrees
Acq. time 1.706 sec
Width 4803.1 Hz
256 repetitions
OBSERVE H1, 300.1988371 MHz
DATA PROCESSING
FT size 16384
Total time 11 min
4f
Agilent Technologies
ppm

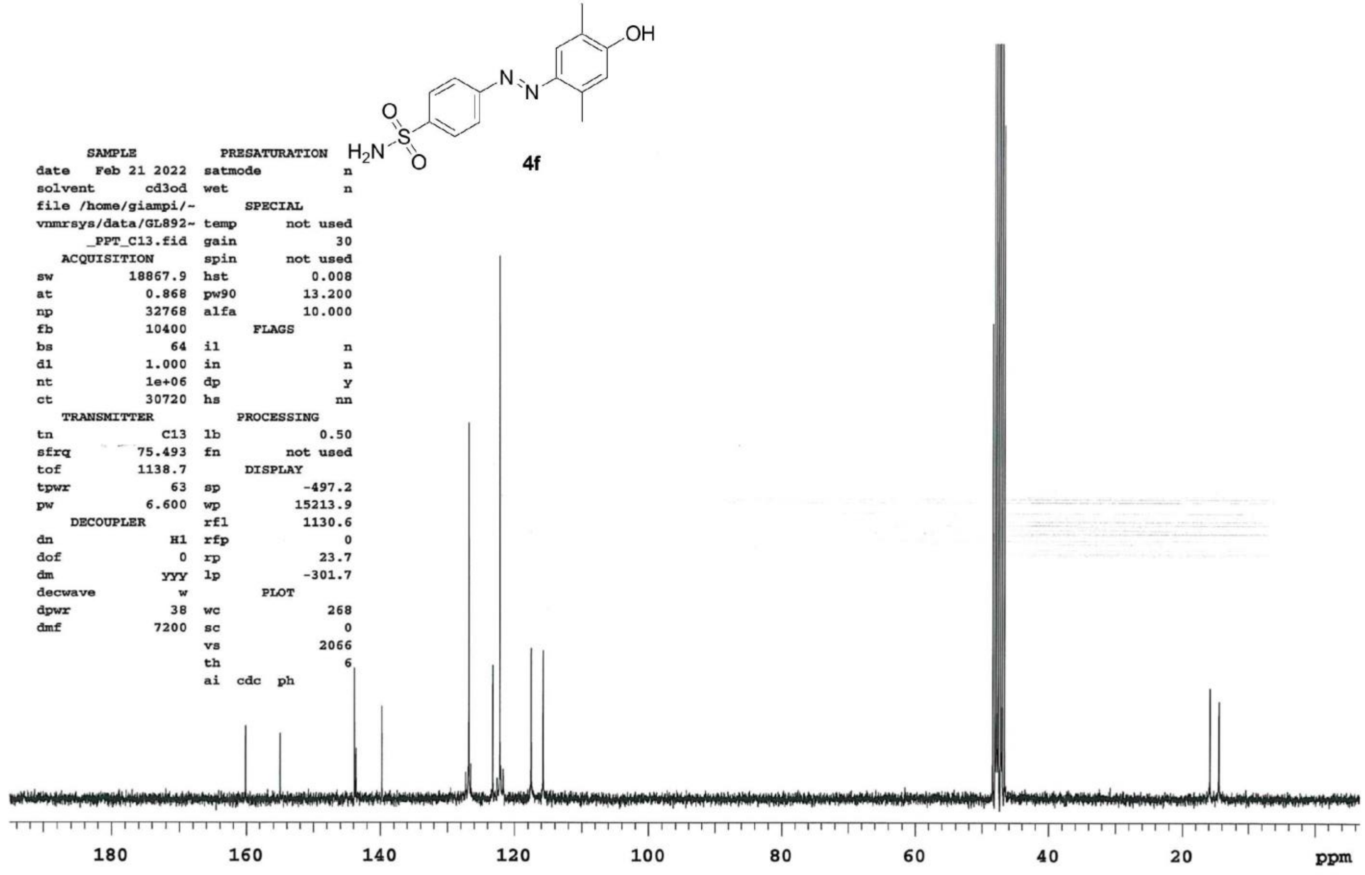
4f
H2N S O O N N OH
SAMPLE
date Feb 21 2022
solvent cd3od
file /home/giampi/~
vnmrsys/data/GL892~
_PPT_C13.fid
ACQUISITION
sw 18867.9
at 0.868
np 32768
fb 10400
bs 64
d1 1.000
nt 1e+06
ct 30720
TRANSMITTER
tn C13
sfrq 75.493
tof 1138.7
tpwr 63
pw 6.600
DECOUPLER
dn H1
dof 0
dm yyy
decwave w
dpwr 38
dmf 7200
PRESATURATION
satmode n
wet n
SPECIAL
temp not used
gain 30
spin not used
hst 0.008
pw90 13.200
alfa 10.000
FLAGS
il n
in n
dp y
hs nn
PROCESSING
lb 0.50
fn not used
DISPLAY
sp -497.2
wp 15213.9
rfl 1130.6
rfp 0
rp 23.7
lp -301.7
PLOT
wc 268
sc 0
vs 2066
th 6
ai cdc ph
180 160 140 120 100 80 60 40 20 ppm

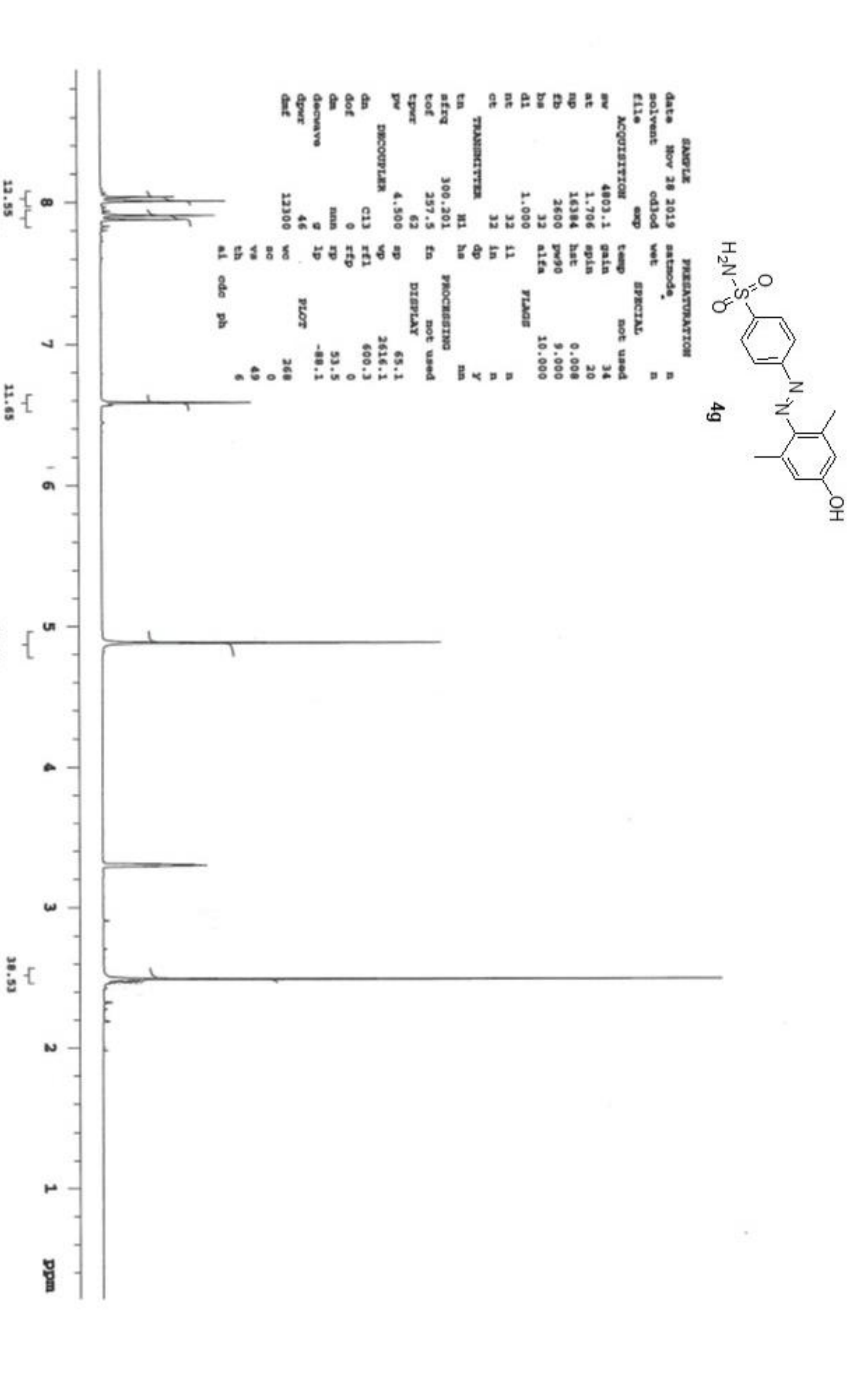
4g
ppm

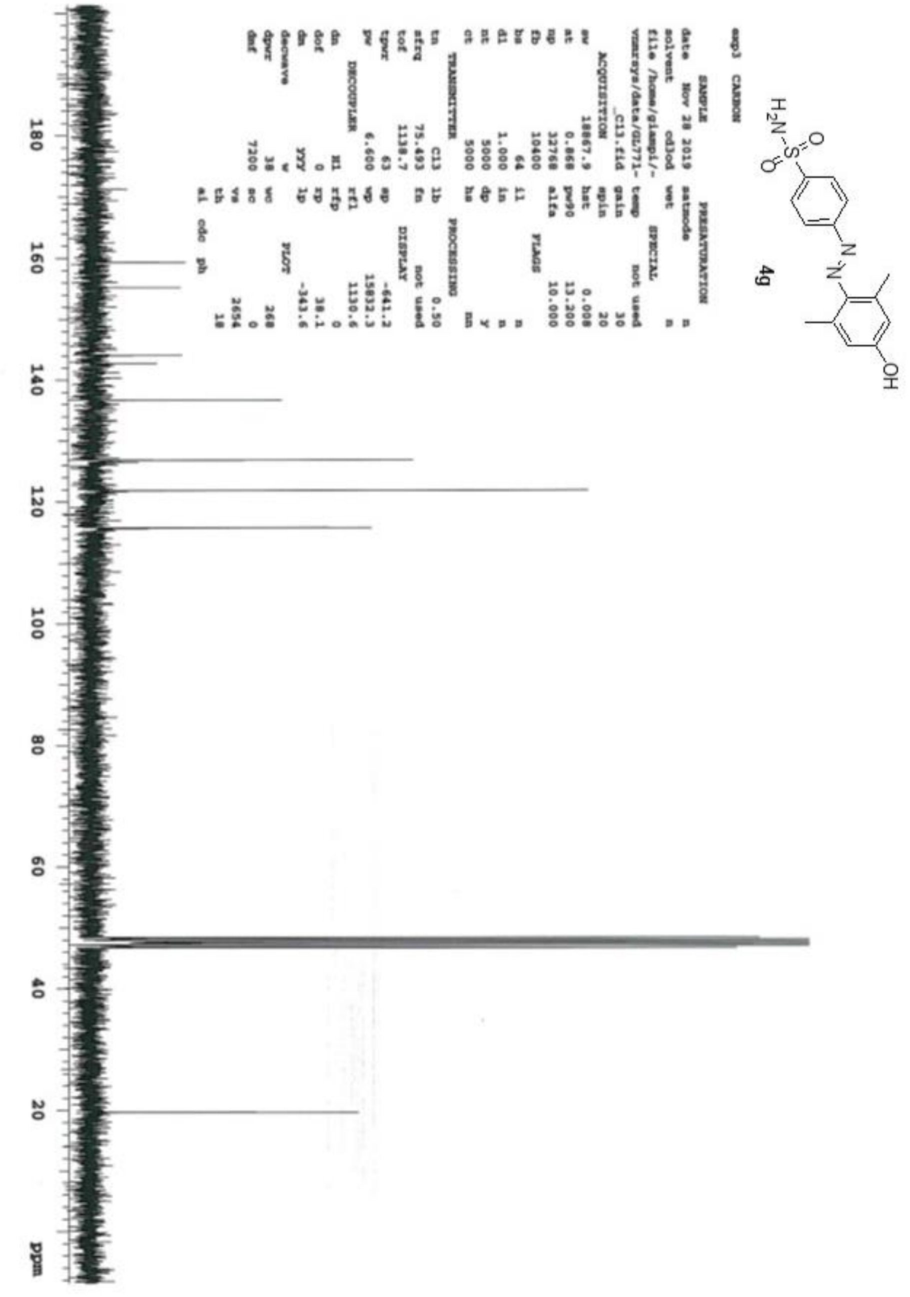

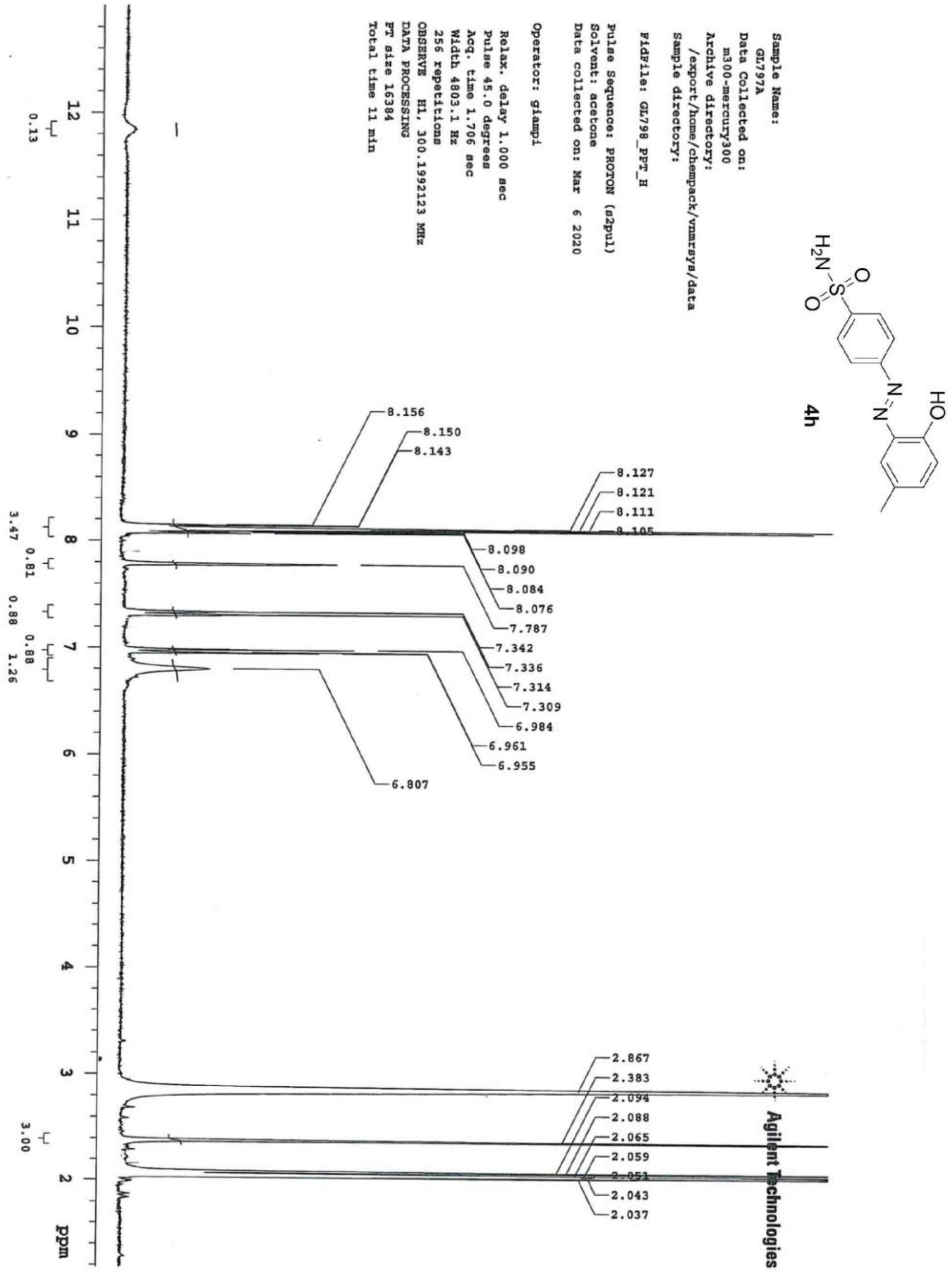

Sample Name:
GL797A
Data Collected on:
m300-mercury300
Archive directory:
/export/home/chempack/vnmrsys/data
Sample directory:
FidFile: GL798_PPT_H
Pulse Sequence: PROTON (s2pul)
Solvent: acetone
Data collected on: Mar 6 2020
Operator: giampi
Relax. delay 1.000 sec
Pulse 45.0 degrees
Acq. time 1.706 sec
Width 4803.1 Hz
256 repetitions
OBSERVE H1, 300.1992123 MHz
DATA PROCESSING
FT size 16384
Total time 11 min
4h
8.156
8.150
8.143
8.127
8.121
8.111
8.105
8.098
8.090
8.084
8.076
7.787
7.342
7.336
7.314
7.309
6.984
6.961
6.955
6.807
2.867
2.383
2.094
2.088
2.065
2.059
2.051
2.043
2.037
0.13
3.47
0.81
0.88
0.88
1.26
3.00
ppm
Agilent Technologies

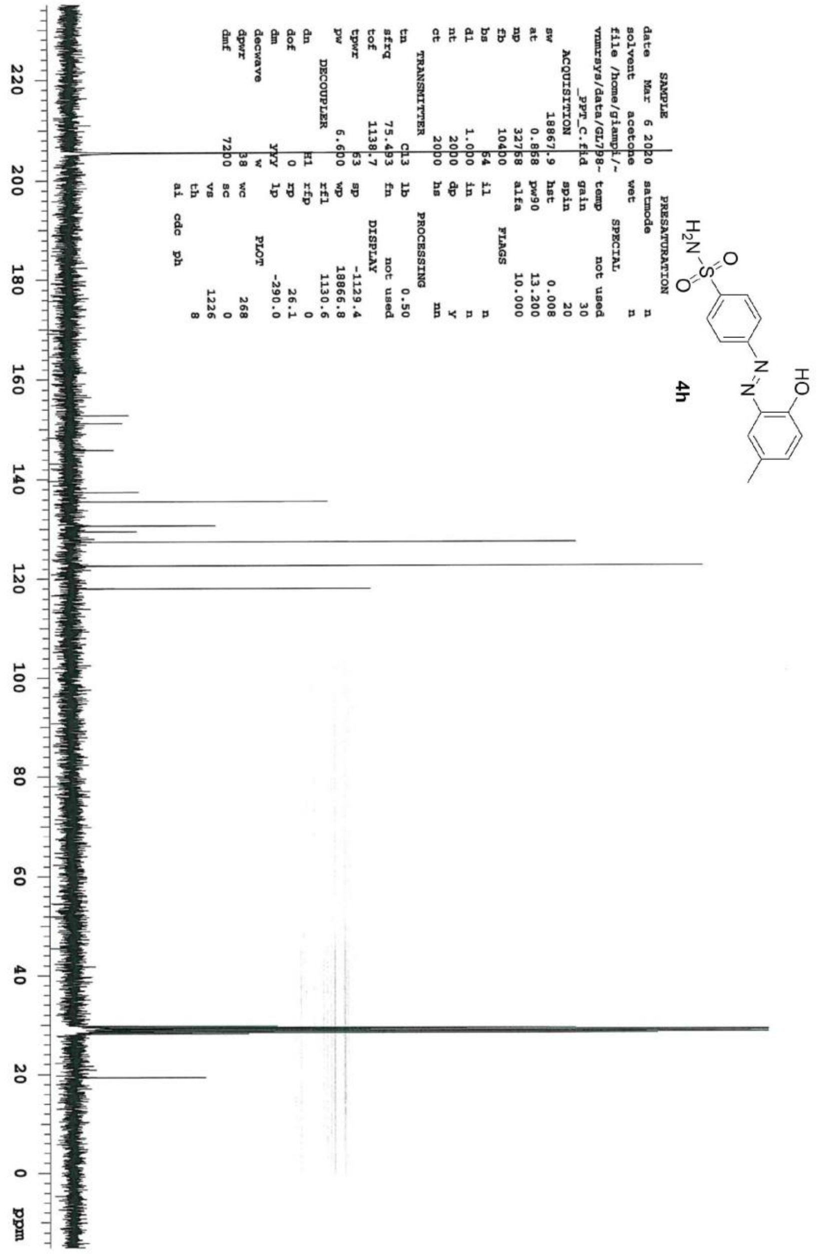
4h
ppm

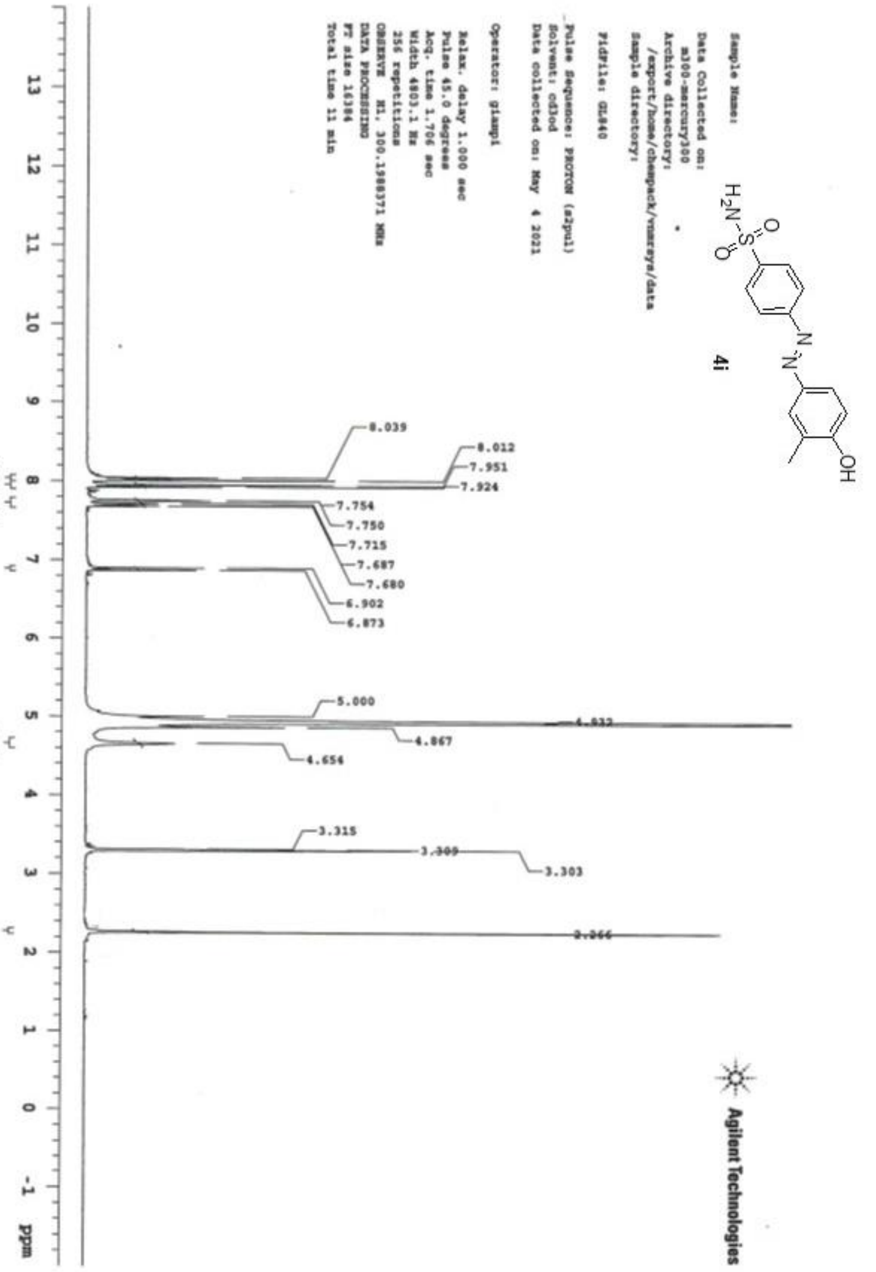
4i
Pulse Sequence: PROTON (s2pul)
Solvent: cd3od
Data collected on: May 4 2021
Agilent Technologies
ppm

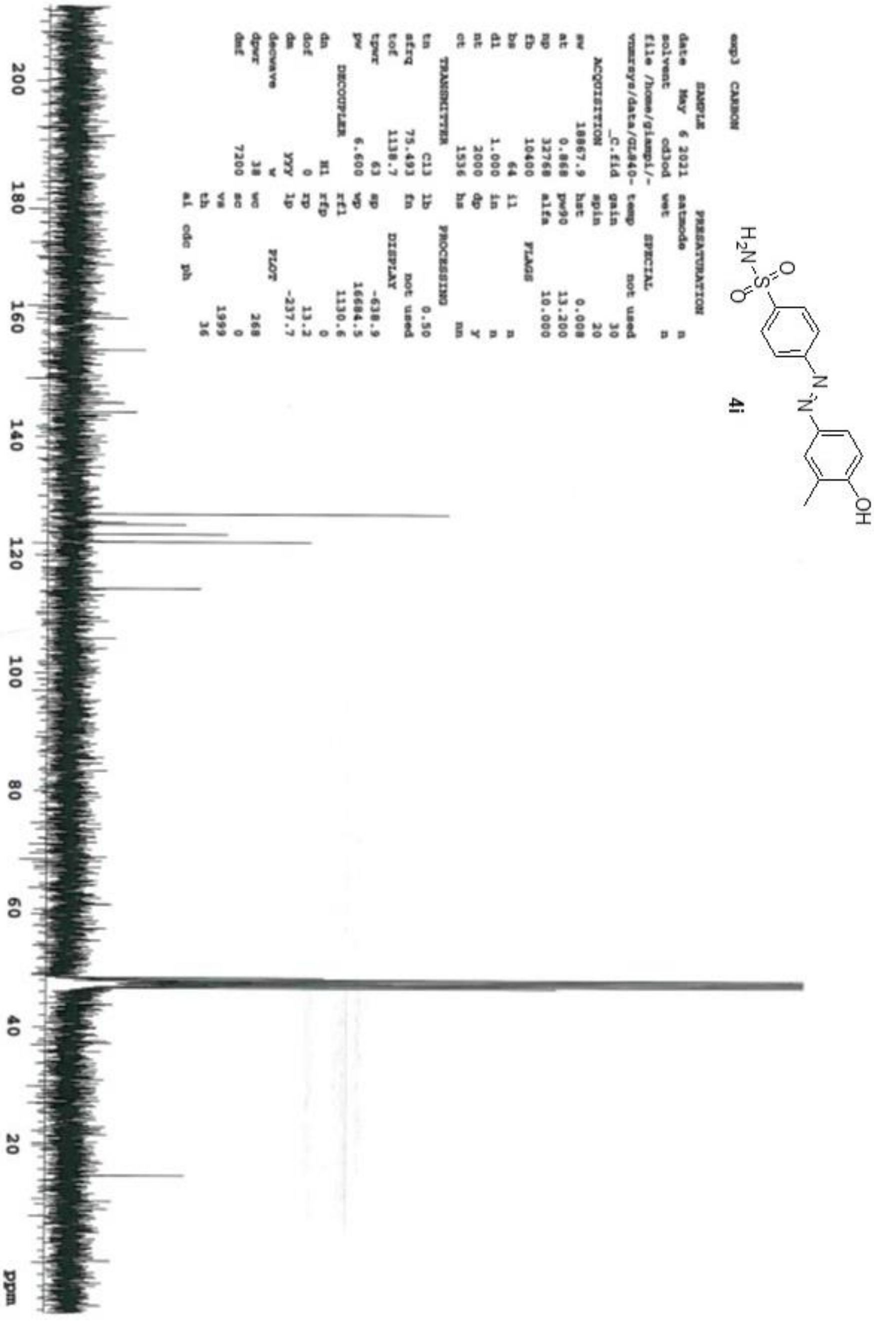

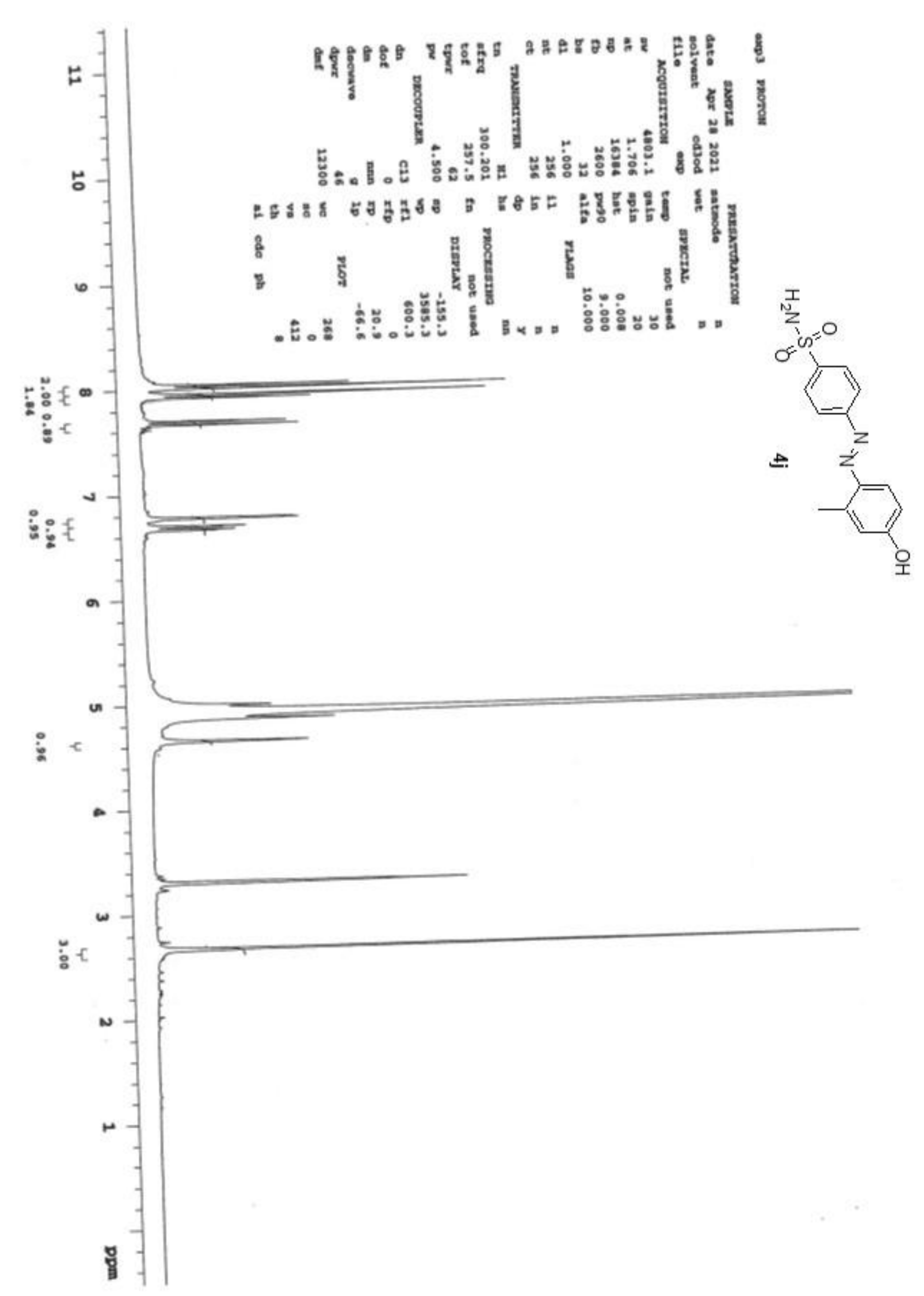

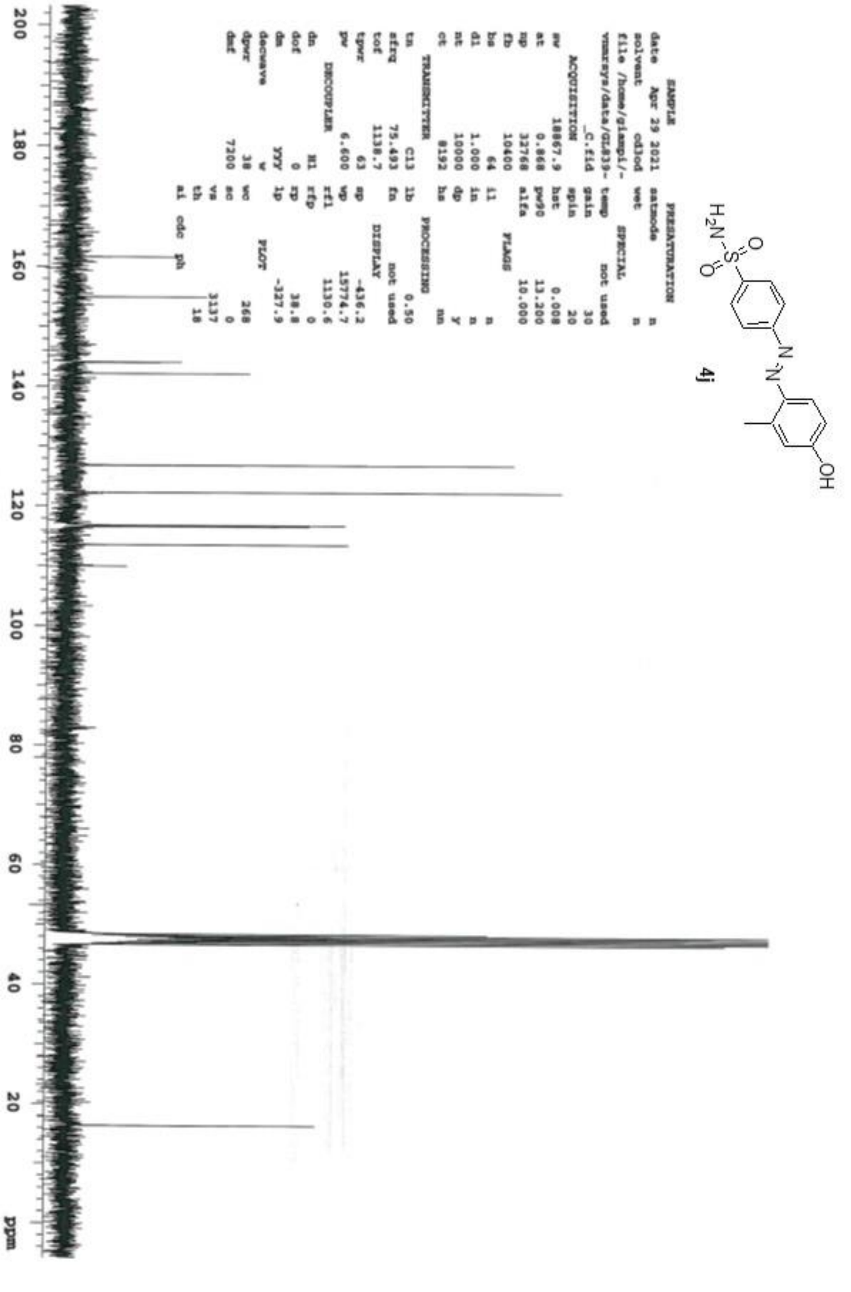

4j

Table S1. Docking scores of compounds **4a-j** to HpCAα and HpCAβ isoforms.

	**DOCKING SCORE (ChemPLP)**	
**Compound**	**HpCAα**	**HpCAβ**
**4a**	73.28	65.39
**4b**	72.1	65.39
**4c**	73.64	66.2
**4d**	75.15	66.22
**4e**	73.25	65.36
**4f**	73.18	65.63
**4g**	72.29	65.59
**4h**	71.74	65.28
**4i**	72.37	65.51
**4j**	72.27	64.34
**R^2 (score *vs* -logK_i)**	0.78	0.67

Even though K_I data are in a narrow range, an attempt to correlate them with computational score was carried out by plotting docking score vs experimental -logK_i values. A very good correlation was obtained, as reported in Table S1, corroborated by R^2 values of 0.78 and 0.67 for HpCAα and HpCAβ, respectively.
